# EAAT3 impedes oligodendrocyte remyelination in chronic cerebral hypoperfusion‐induced white matter injury

**DOI:** 10.1111/cns.14487

**Published:** 2023-10-06

**Authors:** Yingmei Zhang, Dongshan Ya, Jiaxin Yang, Yanlin Jiang, Xiaoxia Li, Jiawen Wang, Ning Tian, Jungang Deng, Bin Yang, Qinghua Li, Rujia Liao

**Affiliations:** ^1^ Laboratory of Neuroscience Affiliated Hospital of Guilin Medical University, Guilin Medical University Guilin China; ^2^ Department of Neurology Affiliated Hospital of Guilin Medical University, Guilin Medical University Guilin China; ^3^ Department of Pharmacology Affiliated Hospital of Guilin Medical University, Guilin Medical University Guilin China; ^4^ Guangxi Clinical Research Center for Neurological Diseases Affiliated Hospital of Guilin Medical University, Guilin Medical University Guilin China

**Keywords:** chronic cerebral hypoperfusion, excitatory amino acid transporter 3 (EAAT3), neuroprotection, oligodendrocyte progenitor cells (OPCs), white matter

## Abstract

**Background:**

Chronic cerebral hypoperfusion‐induced demyelination causes progressive white matter injury, although the pathogenic pathways are unknown.

**Methods:**

The Single Cell Portal and PanglaoDB databases were used to analyze single‐cell RNA sequencing experiments to determine the pattern of EAAT3 expression in CNS cells. Immunofluorescence (IF) was used to detect EAAT3 expression in oligodendrocytes and oligodendrocyte progenitor cells (OPCs). EAAT3 levels in mouse brains were measured using a western blot at various phases of development, as well as in traumatic brain injury (TBI) and intracerebral hemorrhage (ICH) mouse models. The mouse bilateral carotid artery stenosis (BCAS) model was used to create white matter injury. IF, Luxol Fast Blue staining, and electron microscopy were used to investigate the effect of remyelination. 5‐Ethynyl‐2‐Deoxy Uridine staining, transwell chamber assays, and IF were used to examine the effects of OPCs' proliferation, migration, and differentiation in vivo and in vitro. The novel object recognition test, the Y‐maze test, the rotarod test, and the grid walking test were used to examine the impact of behavioral modifications.

**Results:**

A considerable amount of EAAT3 was expressed in OPCs and mature oligodendrocytes, according to single‐cell RNA sequencing data. During multiple critical phases of mouse brain development, there were no substantial changes in EAAT3 levels in the hippocampus, cerebral cortex, or white matter. Furthermore, neither the TBI nor ICH models significantly affected the levels of EAAT3 in the aforementioned brain areas. The chronic white matter injury caused by BCAS, on the other hand, resulted in a strikingly high level of EAAT3 expression in the oligodendroglia and white matter. Correspondingly, blocking EAAT3 assisted in the recovery of cognitive and motor impairment as well as the restoration of cerebral blood flow following BCAS. Furthermore, EAAT3 suppression was connected to improved OPCs' survival and proliferation in vivo as well as faster OPCs' proliferation, migration, and differentiation in vitro. Furthermore, this study revealed that the mTOR pathway is implicated in EAAT3‐mediated remyelination.

**Conclusions:**

Our findings provide the first evidence that abnormally high levels of oligodendroglial EAAT3 in chronic cerebral hypoperfusion impair OPCs' pro‐remyelination actions, hence impeding white matter repair and functional recovery. EAAT3 inhibitors could be useful in the treatment of ischemia demyelination.

## Graphical abstract

We show for the first time that (1) EAAT3 impairs OPC differentiation, migration, and proliferation; (2) EAAT3 suppression for these OPCs aid in the survival of oligodendroglia, remyelination of white matter, and ultimately functional recovery; (3) the phosphorylated mTOR pathway facilitates remyelination and white matter repair through EAAT3 suppression. These results suggest that EAAT3‐targeting strategies may be therapeutically useful for treating demyelinating illnesses.

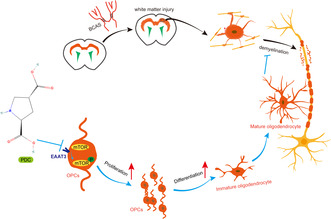

## 1 INTRODUCTION

Aging is a natural process that leads to various changes in the body, including the brain. White matter degeneration, which is linked to a number of cognitive and neurological diseases, is one of the most important alterations that take place in the aging brain.^1^, ^2^ White matter damage can contribute to demyelination, as it leads to axonal degeneration and progressive deterioration in neurological function.^3^, ^4^ Remyelination, on the other hand, can result in the ensheathing of the demyelinated axons with fresh myelin sheaths, which can speed up functional recovery. In the meantime, it stops new nerve fiber injury and can stop neuronal degeneration.^5^, ^6^ Temporarily, some treatments can alleviate the symptoms of demyelination, but more focused therapies are still required to stop myelin breakdown and encourage regeneration.^7^

Oligodendrocytes play a crucial role in the process of remyelination.^8^, ^9^ In demyelinating diseases such as multiple sclerosis or experimental allergic encephalomyelitis, the loss of oligodendrocytes and myelin leads to neurological symptoms.^10^, ^11^ In these circumstances, the differentiation and maturation of oligodendrocyte precursor cells (OPCs) into mature oligodendrocytes can result in the remyelination of injured CNS axons.^12^, ^13^ OPCs move to the location of the lesion during this process, develop into oligodendrocytes, and then cover the exposed axons in fresh myelin.^14^, ^15^ The condition may advance in some situations, though, if the remyelination process fails. This failure may be brought on by insufficient or absent stimulation of OPCs, which may not develop into mature oligodendrocytes and worsen neurological conditions.^16^, ^17^ Therefore, enhancing OPCs' proliferation, migration, and differentiation may repair hypermyelination abnormalities and lead to better clinical outcomes. But there are not many pharmacological targets that can be affected to promote OPCs' migration, proliferation, and differentiation.

Excitatory amino acid transporter 3 (EAAT3, encoded by SLC1A1), as a member of EAATs family, plays a critical role in the maintenance of neurotransmission and preventing excitotoxicity, which have been extensively reviewed previously,^18^, ^19^ while EAAT3 was previously found in the post‐synaptic terminals of most neurons in the CNS. Through the specific transporters EAAT3, neurons contribute efficiently to the clearance of excitatory amino acids.^20^, ^21^ Numerous studies have recently focused on the possible fine tuning of EAAT3, which is related either to neuronal activity or to selective activation of post‐synaptic signaling pathways acting through post‐translational mechanisms, emphasizing the dynamic character of the neuronal uptake process.^22^, ^23^ In this respect, it is interesting to note that EAAT3 exhibits specificity, compared with other EAA transporters, because it is present mainly in the intracellular compartment and only for about 20% at the plasma membrane.^24^ Thus, EAAT3 could be involved in other roles independent of its contribution to EAA synaptic removal. It is interesting to note that a recent study found that increasing EAAT3's absorption of cysteine might maintain the brain's OPCs' ability to differentiate. In addition, EAAT3‐deficient mice have shown age‐dependent neurodegeneration and decreased number of axons in the corpus callosum, further suggesting that EAAT3 plays a potential role in oligodendrocytes and remyelination.^25^

In this study, we set out to investigate the potential role of EAAT3 in chronic white matter damage. We were particularly interested in its possible role in the remyelination process. A specific remarkable increase in oligodendroglial EAAT3 expression was accompanied by white matter demyelination. The EAAT3 inhibition with a specific antagonist ameliorated the demyelination and improved cognitive and motor dysfunction in the neuro‐function. EAAT3 inhibition promoted OPCs' proliferation, migration, and differentiation via the mTOR signaling pathway, and it also promoted oligodendrocytes myelination. These findings, collectively, reveal a critical role of EAAT3 in the regulation of oligodendroglial function and demyelination following white matter injury.

## 2 MATERIALS AND METHODS

### 2.1 Animals

Healthy male C57BL/6j mice weighing between 25 and 30 g were obtained from Hunan SJA Laboratory Animal Co., Ltd. and housed in individually ventilated cages under a 12:12‐h light/dark cycle with free access to food and water. The mice were kept in a room maintained at 50% humidity and approximately 25°C. All animal trials were approved by the Animal Experimentation Committee of Guilin Medical University (approval number GLMC202103063). The mice were randomly assigned to the sham‐operated group, the chronic cerebral hypoperfusion model group, and the L‐trans‐2,4‐pyrrolidine dicarboxylate (PDC)‐treated group. In order to limit any pain or distress, the fewest number of animals was used as possible. One hundred and eighteen mice were used in this study. Mice were randomly divided into different groups: Sham group (*n* = 24); intracerebral hemorrhage model (ICH) group (*n* = 4, 1 mouse died, respectively); traumatic brain injury model (TBI) group (*n* = 4); 2‐week‐old naive mice group (*n* = 3); 4‐week‐old naive mice group (*n* = 3); 6‐week‐old naive mice group (*n* = 3); 8‐week‐old naive mice group (*n* = 3); 1 day after bilateral common carotid artery stenosis (BCAS) group (*n* = 7, 1 mouse died, respectively); 7 days after BCAS group (*n* = 7); 14 days after BCAS group (*n* = 23, 2 mice died, respectively); 28 days after BCAS group (*n* = 7, 1 mouse died, respectively); BCAS + PDC group (*n* = 18, 3 mice died, respectively); sham + EdU group(*n* = 3); BCAS + EdU group (*n* = 3); BCAS + PDC+ EdU group (*n* = 3); BCAS + PDC+ Rap group (*n* = 3). Besides this, we found no significant difference in the change in body weight of the experimental animals over the course of the experiment.

### 2.2 Bilateral common carotid artery stenosis surgery

Bilateral common carotid artery stenosis surgery was used as a chronic cerebral hypoperfusion mouse model as previously reported.^26^ The experiment involved surgical mice that were fixed to a surgical table equipped with a gas anesthesia system (R500, RWD Life Science). The anesthesia was induced by administering an anesthetic dosage of 3.5 mL/min and adjusting the air flow meter to 3–4 L/min. Once the mouse was completely unconscious, the anesthetic concentration was reduced to 1–1.5 mL/min, and the gas flow meter was adjusted to 0.5 L/min to maintain the procedure. Isoflurane was the anesthetic used in this experiment. A longitudinal incision was made from the center of the neck after preparing and cleaning the skin of the surgical site. The subcutaneous tissue was carefully and gently dissected under a microscope. Micro‐curved forceps were then used on one side to hold up the common carotid artery, while on the other side, another micro‐curved forcep was used to quickly spin a small spring coil with a 0.18 mm inner diameter from the carotid bifurcation's lower end to the common carotid artery. The surgeon should be cautious throughout the procedure to avoid piercing the common carotid artery with the tip of the spring. After completing the surgery, the incision was sutured, and the area was cleaned. The rectal temperature was maintained between 36.5 and 37.5°C during surgery. After this procedure, the mice were observed for 2 h in a temperature‐controlled incubator until they became conscious and recovered enough to freely access food and water ad libitum. Sham animals were given an incision in the skin, and their common carotid arteries were exposed.

### 2.3 Traumatic brain injury model

Controlled cortical impact (CCI) to the right hemisphere of the brain was used to cause TBI in adult mice aged 8 weeks.^27^ Sevoflurane 2% was used to quickly produce anesthesia in mice. In order to reveal the skull, the mouse's head was cut open and stabilized in a stereotaxic frame. To expose the dura and cerebral cortex, a right parietal craniotomy (centered 0.5 mm anterior and 2.0 mm lateral to the bregma; diameter: 3.5 mm) was made. The CCI procedure was carried out using pneumatically powered CCI equipment (68099II; RWD Life Science) that compressed the exposed brain tissue to a depth of 1.5 mm (dwell time: 150 ms; peak velocity: 3.5 m/s).

### 2.4 Intracerebral hemorrhage model

With 2% isoflurane, adult mice aged 8 weeks were put to sleep. A stereotactic frame was used to position the mice. A 1‐mm burr hole was drilled into the skull. Type IV collagenase (C4BIOC; Sigma) was slowly injected intracranially into the right basal ganglia using a micro‐infusion pump (R462; RWD Life Science) at a rate of 0.05 L/min to cause intracerebral bleeding (coordinates: 0.1 mm anterior, 1.0 mm posterior, and 3.0 mm depth). Instead of IV collagenase, 0.9% saline was administered for the sham group.^28^

### 2.5 Drug administration

After BCAS surgery, mice were intracerebroventricular (AP: −0.5 mm, L: −1.0 mm, V: −2 mm) injected with saline or L‐trans‐2,4‐pyrrolidine dicarboxylate (PDC, P7575; Sigma; 0.8 μg in 2 μL saline per day) continuously for 14 days.^29^ To study cell proliferation, mice were injected with EdU (5 mg/kg, in saline, C00053; RiboBio Co., Ltd.) for seven consecutive days following the modeling, with a 12‐h gap between each injection.^30^ Animals were injected with rapamycin (Rap, AY‐22989; MCE) at a dose of 10 mg/kg once a day for 14 days.

### 2.6 Bioinformatics analysis of the single‐cell sequencing dataset

The single‐cell RNA sequencing data and the information on the corresponding annotation were retrieved from the Single Cell Portal (https://singlecell.broadinstitute.org/single_cell) and PanglaoDB (https://panglaodb.se/). We searched the data of Single Cell Portal using the key term “slc1a1” and discovered the study “A single‐cell map of antisense oligonucleotide activity in the brain.” The visualization of UMAP plots was derived from the above study. We searched PanglaoDB for the term “slc1a1” and downloaded the “Summary of search results” as a tsv file. Then, using these data, we created a display of annotated cell clusters.

### 2.7 Laser speckle cerebral blood flow (CBF) testing

The mice were initially anesthetized with 2% sevoflurane. Their heads were then cleaned, and the skin was sliced along the midline of the skull to reveal the mouse skull. The exposed portion of the skull was subsequently covered with saline drops, and a laser scatter blood flow detection device (RFSLI ZW/RFLSI III, RWD Life Science, Shenzhen, China) was used to monitor the CBF. A camera positioned 10 cm above the skull was used to capture raw speckle images of areas of interest (ROIs) covering the parietal lobe in each hemisphere. At pre, ~5 min, 1 day, 7 days, and 14 days after operation, and for the same‐sized regions in both parietal lobes, regional CBF values (arbitrary perfusion units) were assessed. By comparing the mean signal intensity to the baseline, it was possible to determine the percentage change in regional CBF at each post‐surgery time point. Because isoflurane administration may have impacts on CBF according to prior research, we kept the isoflurane treatment time and concentration the same for each animal to avoid any possible confounding effects in each group.^31^, ^32^

### 2.8 Luxol fast blue (LFB) staining

Mouse brain tissue was taken and fixed in 4% paraformaldehyde for 48 h. A frozen sectioning machine (Cryostar NX50; Thermo Scientific) was used to cut the tissue into 10‐μm‐thin coronal brain slices for later staining after the tissue had been dried with paraformaldehyde containing 30% sucrose. Before staining, brain slices were washed with PBS for 5 min, double‐distilled water for 5 min, and then graded ethanol (75%, 95%, 100%) for 2 min for dehydration. The slices were immersed in LFB staining solution (G3245; Beijing Solarbio Science & Technology Co., Ltd.) and kept at room temperature for an entire night. The extra floating color was treated with 95% ethanol the next day, then washed with double‐distilled water to eliminate it. Slices of the brain were submerged in LFB differentiation solution for 15 s and 70% ethanol for 30 s. The gray matter and white matter were clearly distinguished after microscopically observing the staining elution. Sections were re‐stained with a tar violet staining solution for 30 to 40 seconds, and then, double‐distilled water was used to rinse them. The sections underwent a gradient of 95% to100% ethanol dehydration. The parts were then made transparent with xylene and sealed with neutral resin. Using a Leica microscope (Leica Dm2500; Leica Co.), bright‐field pictures were captured. As previously mentioned, the corpus callosum (Paramedian), corpus callosum (Medial), and caudoputamen were examined for white matter abnormalities. White matter lesions were rated according to their severity (severity index) as normal (grade 0), nerve fiber disarray (grade 1), significant vacuole development (grade 2), and myelinated fiber loss (grade 3) as previously described.^26^ The evaluation of severity index was calculated by blinded independent readers.

### 2.9 Immunofluorescence analysis

Frozen tissue sections used for immunofluorescence staining were identical to the LFB‐stained sections described above. Briefly, tissue sections and cells were washed three times with PBS for 5 min each time and then blocked for 2 h with a PBS solution containing 10% goat serum. They were then treated with the primary antibodies listed below for mouse anti‐CNPase (1:200, 66729‐1‐Ig; Proteintech), rabbit anti‐NG2 (1:200, DF12589; Affinity Biosciences), rabbit anti‐MBP (1:200, AF4085; Affinity Biosciences), mouse anti‐Olig2 (1:200, 66513‐1‐Ig; Proteintech), rabbit anti‐Olig2 (1:200, 13999‐1‐AP; Proteintech), mouse anti‐P‐mToR (1:200, 67778‐1‐Ig; Proteintech), mouse anti‐A2B5 (1:200, ab53521; Abcam), and rabbit anti‐EAAT3 (1:200, ab288441; Abcam) overnight at 4°C. The following day, sections and cells were washed in PBS and incubated with Alexa Fluor‐conjugated 488 or 594 secondary antibodies to rabbit or mouse (1:200; Affinity Biosciences) for an hour at room temperature. After removing the secondary antibody with PBS, anti‐fluorescence attenuation blocker (S2110; Beijing Solarbio Science & Technology Co., Ltd.) was used to block the sections and cells. The sections were then captured in photographs using a fluorescent microscope. All images were analyzed using ImageJ software. The analysis of immunofluorescence images was calculated by blinded independent readers.

### 2.10 5‐Ethynyl‐2‐deoxy uridine (EdU) staining

The brain was taken after perfusion with paraformaldehyde 2 h after the last injection, cut into 16‐μm slices, and left aside. Following staining with additional primary and secondary antibodies, sections were washed three times for 10 min in PBS, then permeabilized for 20 min in 0.3% Triton X‐100, washed again three times, and then stained following the Cell‐light™ Apollo 567 Stain Kit (C10371‐1; RiboBio Co., Ltd.). For cells staining, they are incubated 2 h in advance in medium containing 50 μM EdU, then fixed and subsequently stained in accordance with the tissue sectioning procedure.

### 2.11 Western blotting

On ice, isolated portions of the brain were homogenized using 500 μL of RIPA Protein Lysate and 5 μL of PMSF Protease Inhibitor. The suspension was placed on ice and lysed for 30 min. Samples of brain protein were collected for detection. Electrophoresis (SDS‐PAGE) was used to separate equal amounts of proteins on a 7.5–12% sodium dodecyl sulfate‐polyacrylamide gel, which was then transferred to a PVDF membrane. Membranes were blocked with a TBST solution containing 5% non‐fat milk powder for an hour. The blocking solution was washed off and probed with primary antibodies including mouse anti‐CNPase (1:1000, 66729‐1‐Ig; Proteintech), rabbit anti‐NG2 (1:1000, DF12589; Affinity Biosciences), rabbit anti‐MBP (1:1000, AF4085; Affinity), mouse anti‐P‐mTOR (1:1000, 67778‐1‐Ig; Proteintech), rabbit anti‐mTOR (1:1000, 2983; CST), rabbit anti‐EAAT3 (1:1000, ab288441; Abcam), mouse anti‐GAPDH (1:2000, 60004‐1‐Ig; Proteintech), and rabbit anti‐β‐actin (1:5000, 4970; CST) overnight at 4°C on a shaker. The next day, the horseradish peroxidase‐conjugated anti‐mouse (1:2000; CST) or anti‐rabbit antibody (1:2000; CST) was incubated for 1 h. An enhanced chemiluminescence detection system (P0018S; Beyotime Biotech) was used to detect the bands. ImageJ was used to examine the bands (Figure S4).

### 2.12 Novel object recognition test

During the adjustment period, mice were allowed to freely explore the test device for 15 min. During the training phase, two identical objects were placed in the space, and the mice were allowed to explore them for 5 min. One of the recognizable items was randomly selected and replaced with a new one during the testing period an hour later, and the mice were then allowed to explore for an additional 5 min. Before each animal was examined, the box was cleaned with 70% alcohol to prevent interference from the sense of smell. We noted how much time the mice spent investigating an object when they displayed sniffing behavior toward it and were within 2 cm of it. It is preferable to avoid remaining motionless while sitting or standing, though. The amount of time the mice spent on the gadget during the allotted time for various tasks was investigated using video capture and statistical analysis. The percentage of exploratory preference was calculated by dividing the amount of time spent researching a new object by the total amount of time spent exploring both objects. All behavioral tests and data analysis were conducted in a blind manner.

### 2.13 Y‐maze test

The Y‐maze is used to test spatial cognition and memory functions. As previously described, it consists of three equally sized arms that are at a 120‐degree angle to each other (Xinruan Instruments).^33^ Different geometric shapes were placed inside each arm of the maze to serve as visual cues during the experiment. The experiment consisted of two phases. In the first phase, the mouse is placed with its back to the center of the maze, with one of the arms (the new arm) blocked by a divider. It is then given 10 min to explore the initial arm and the remaining arms before being removed. The second phase of the test period begins after a 1‐h break. The neoisolation arm is opened, the rat is still in the initial arm, and it spends 5 min exploring the three arms. It is noted how often the animal enters each arm and how long it stays there. All behavioral tests and data analysis were conducted in a blind manner.

### 2.14 Rotarod test

The test was designed to evaluate motor balance and coordination in mice. The experimental mice were mounted on an accelerating rotarod cylinder (Xinruan Instruments), and the rotational speed of the cylinder was regulated to increase progressively from 0 to 40 rpm within 1.5 min, as described in our previous research. During the procedure, the latency to fall was recorded. All behavioral tests and data analysis were conducted in a blind manner.

### 2.15 Grid walking test

For the grid walking test, a square metal grid was utilized. The test began with the mouse being placed on top of the grid. The mouse was then given full reign to explore the apparatus, while its behavior was recorded to determine any stepping problems (foot faults). For each limb, the combined number of foot faults and non‐foot‐fault steps was calculated. The number of foot faults was divided by the sum of the number of foot faults and non‐foot‐fault steps, multiplied by 100, to determine the ratio of foot faults to total steps. All behavioral tests and data analysis were conducted in a blind manner.

### 2.16 Electron microscopy

After the mice were deeply anesthetized, the brain tissue was quickly removed, and the corpus callosum was separated from the ice. The tissue was immediately fixed in 2.5% glutaraldehyde (P1126; Beijing Solarbio Science & Technology Co., Ltd.), and a 1‐mm cube tissue block was obtained from the corpus callosum of the mice. Then, the tissue was quickly placed in 2.5% glutaraldehyde for incubation at 4°C overnight, and then fixed with 2% osmium tetroxide in PBS. After dehydration through an alcohol gradient, the samples were embedded in epoxy resin. Semi‐thin sections were then examined by transmission electron microscopy. The G‐ratio, which refers to the diameter of the axon/the diameter of the entire myelinated fiber, is used as an indicator.^34^

### 2.17 Oligodendrocyte progenitor cells culture

Primary cortical OPCs were prepared and maintained as described previously.^35^ Briefly, cells were dissociated from the cortex of neonatal mice after 1–3 days. Dissociated cells were plated in 75‐cm^2^ flasks coated with 0.01% Poly‐D‐lysine (A3890401; Gibco) and maintained in DMEM containing 20% heat‐inactivated fetal bovine serum and 1% penicillin/streptomycin. After 10–12 days of inoculation, cells were placed on an orbital shaker at 37°C and shaken at 200 rpm for 1 h to remove microglia. The medium was then replaced with fresh medium and allowed to stand for 4 h before being shaken horizontally at 220 rpm for 16–18 h. The suspension was collected and left in the incubator for 30 min to remove astrocytes and microglia. The non‐adherent cells were collected and replanted in neurobasal medium containing glutamine, 1% penicillin/streptomycin, 10 ng/mL PDGF‐AA (100‐13A; Peprotech), 10 ng/mL FGF (AF‐100‐18B; Peprotech), 2% B27 (17504044; Gibco), and 1% N2 (17502048; Gibco) supplement on poly‐DL‐ornithine‐coated plates. Four to five days after plating, the OPCs were used for experiments. For cell differentiation, PDGF‐AA and FGF growth factor were excluded from the OPCs medium, and 3,3′,5‐triiodo‐L‐thyronine (T3; 40 ng/mL; Sigma‐Aldrich) and ciliary neurotrophic factor (CNTF; 10 ng/mL; MCE) were added to allow their differentiation.

### 2.18 SYTOX fluorescence staining

High‐affinity SYTOX nucleic acid stain (S11348; Thermo Scientific) will not pass the membranes of living cells but is able to infiltrate cells with damaged plasma membranes with ease. In a nutshell, the 5 μM SYTOX‐blue fluorescent probe was incubated with all cells for 5 min at ambient temperature. Under the fluorescence microscope, images in bright field and SYTOX‐blue fluorescent were captured.

### 2.19 Transwell chamber assay

The OPCs migration experiments were carried out using a transwell‐based Boyden chamber device with an 8‐μm pore size (Corning Incorporated). Purified OPCs were inoculated at a density of roughly 20,000 cells/well in the upper transwell chamber and allowed to migrate for 24 h after both sides of the transwell membrane were pre‐coated with Poly‐D‐lysine. The bottom transwell chamber then received standard OPCs media and medium with PDC. The non‐migrated cells in the top chamber were cleaned with a cotton swab, and the cells that had migrated were stained with a 10% crystal violet staining solution for 5–10 min. The cells were then preserved with 4% PFA for 10 min. The migrating cells were counted under the microscope in eight randomly chosen fields of view from each well after the PC membranes of the chambers had been rinsed until there was no longer any discernible purple color.

### 2.20 Sholl analysis

Cellular immunofluorescence anti‐MBP labeling of oligodendrocytes served to evaluate morphology by counting the number of radium interceptions and the intricacy of the process branching. The diameter of the cell was calculated as its circumference up to its longest projection, measured in distance. The number of crossings that processes made with concentric circles—which are numbered 1–3 to signify increasing distance from the cell body—was used to quantify branching. According to the developers' directions, the quantification was carried out using the ImageJ Sholl Analysis Plugin (NIH; created by Wayne Rasband).^5^

### 2.21 Transfection

OPCs were plated to 70% confluence, at 24 h, cells were serum‐starved for 6 h, then transfected with Small Interfering RNA (siRNA; Sangon Biotech Co., Ltd.) under the protocol of the supplier using Lipofectamine 3000 Reagent (Thermo Fisher Scientific). The following sequences were used to produce siRNAs for mouse EAAT3: (sense, CGUGGUACUAGGAAUUGUCUUTT; antisense, AAGACAAUUCCUAGUACCACGTT). The sequences of siNC are as follows: (sense, UUCUCCGAACGUGUCACGUTT; antisense, ACGUGACACGUUCGGAGAATT).

### 2.22 Statistical analysis

All data were collected and analyzed in a blind manner. With the software program GraphPad Prism 8.0.2, statistical analysis was carried out (GraphPad Software). The mean and standard error (SEM) are used to represent all results. All data used for analysis were normally distributed using the Shapiro–Wilk test. Statistical differences between the two groups were compared using the Student *t* test, while non‐normally distributed variables were compared by the Mann–Whitney *U* test. For multiple comparisons of more than two groups, data were analyzed using one‐way analysis of variance (ANOVA) followed by Bonferroni's post‐hoc test with normally distributed or by the Kruskal–Wallis test with non‐normally distributed. Statistics were deemed significant at *p* < 0.05.

## 3 RESULTS

### 3.1 Aberrant high expression of oligodendroglia EAAT3 after chronic white matter injury

**FIGURE 1:**
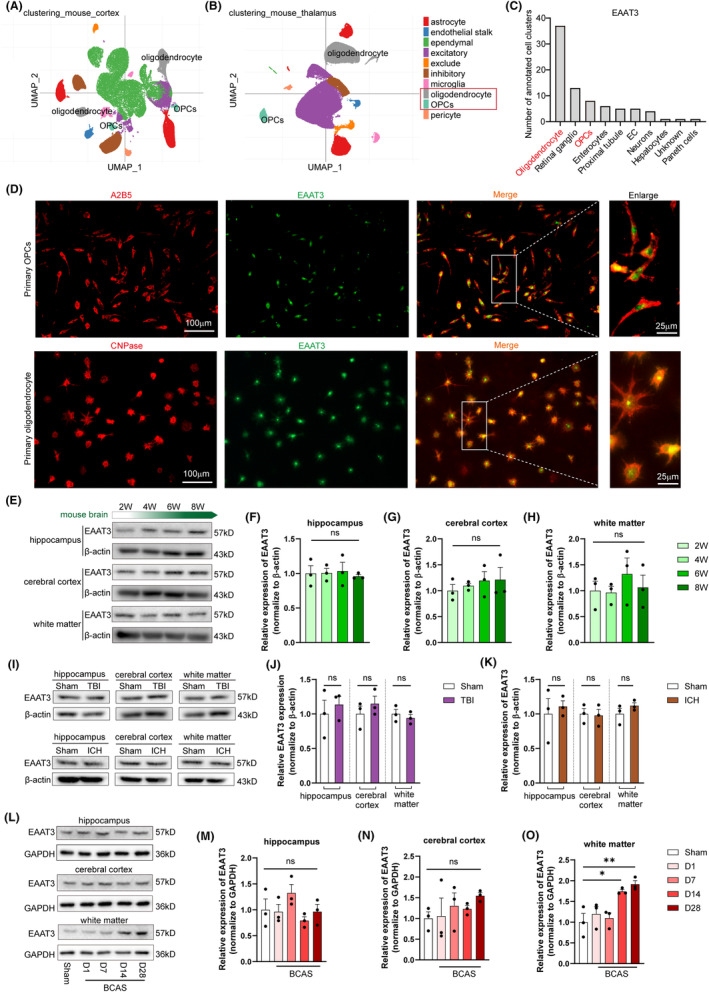
High expression of EAAT3 after white matter injury. UMAP plot showing clusters and cell type annotations in mouse cortex (A) and thalamus (B) in the Single Cell Portal database. (C) the EAAT3 expression in annotated cell clusters in the PanglaoDB database. (D) Representative immunofluorescence image and enlarged confocal image of A2B5 (red) and EAAT3 (green) in primary OPCs, as well as CNPase (red) and EAAT3 (green) in primary oligodendrocytes. (E–H) Representative immunoblotting images and quantification of relative protein level of EAAT3 in the hippocampus, cerebral cortex, and white matter at weeks 2, 4, 6, and 8 of mouse brain development, *n* = 3 in each group. (I–K) In a mouse model of traumatic brain injury and intracerebral hemorrhage, representative immunoblotting pictures and quantification of the relative protein level of EAAT3 in the hippocampus, cerebral cortex, and white matter were performed on Day 1, *n* = 3 in each group. (L–O) Representative western blot images and quantification of EAAT3 expression in the white matter, hippocampus, and cerebral cortex at different days after BCAS mice, *n* = 3 in each group. There was no difference in body weight between mice in each group. The data for each group conformed to a normal distribution. The *p* value was determined by ANOVA with Bonferroni's post‐hoc test. *p < 0.05, **p < 0.01, between the indicated groups, ns indicates non‐significance. Data are represented as means ± SEM.

We initially attempted to evaluate the expression pattern of EAAT3 in central nervous system (CNS) cells. A considerable fraction of EAAT3 is expressed on neurons, according to data from publicly available single‐cell RNA sequencing studies in the Single Cell Portal (Figure 1A,B) and PanglaoDB (Figure 1C) databases, but intriguingly, a significant portion of EAAT3 is also expressed on oligodendrocyte lineages, including oligodendrocyte precursor cells (OPCs) and mature oligodendrocytes, which has not previously been documented. Next, the studies of immunostaining for EAAT3 in primary OPCs and primary oligodendrocytes validated that EAAT3 is expressed by the majority of oligodendrocyte lineages (Figure 1D). The function of EAAT3 in oligodendrocyte lineages was then something we wanted to further assess. We initially investigated the levels of EAAT3 at several phases of mouse brain development since oligodendrocyte lineages are important for brain development. Sadly, the results revealed that there were no significant changes in EAAT3 levels in the hippocampus, cerebral cortex, and white matter during numerous crucial periods of mouse brain development, including 2, 4, 6, and 8 weeks (Figure 1E–H). Using mice models of traumatic brain injury (TBI) and intracerebral hemorrhage (ICH) as models of acute brain injury, which might result in neuronal and oligodendrocyte damage in the brain during the acute phase, we further explored whether EAAT3 has a role in the status of CNS diseases. The findings demonstrated that neither of the acute brain damage models significantly altered the levels of EAAT3 in the hippocampus, cortex, or white matter (Figure 1I–K). These findings imply that in cases of acute brain damage, EAAT3 has little impact on neurons or oligodendrocytes. As a result, we looked more closely at how EAAT3 affected neurons with oligodendrocytes in the setting of chronic brain injury.

In order to imitate a normal and clinically prevalent chronic brain injury paradigm, we further employed a chronic cerebral hypoperfusion‐induced mouse brain damage model. This model can cause long‐term harm to both the oligodendrocytes and neurons. We adopted the BCAS mice model of chronic hypoperfusion, in which coils are permanently placed around both common carotid arteries as previously reported (Figure S1A). We further examined the expression of EAAT3 after BCAS. It is important to note that our findings revealed that, since 14 days BCAS, compared to sham controls, the expression of EAAT3 in the white matter was much higher (Figure 1L,O). Concurrently, the expression of EAAT3 exhibited no detectable changes in both the hippocampus (Figure 1L,M) and cerebral cortex (Figure 1L,N). These results indicate that while EAAT3 levels in neurons remain unchanged, EAAT3‐specific high expression is displayed in the white matter region where oligodendrocytes gather in the state of chronic brain damage.

The pathological alterations in the white matter location following chronic brain damage in BCAS mice were next studied. The hallmark phenotypes of white matter lesions in the BCAS mice were examined using a mature oligodendrocyte marker 1 day, 7 days, 14 days, and 1 month following induction. Correspondingly, as established by CNPase western blots analysis, the expression level of CNPase, an oligodendrocyte marker, demonstrates an overall declining tendency at 1 day and 7 days, a substantial drop at 14 days, and lasted until 1 month post‐BCAS in white matter (Figure S1B,C). Immunofluorescence also corroborated the alteration in CNPase levels in the corpus callosum and revealed that the number of CNPase‐positive cells was considerably reduced 14 days after BCAS (Figure S1D,E). The myelin integrity of two corpus callosum regions (paramedian and medial) and causoputamen was then assessed using LEB staining. The callosum regions and causoputamen of sham controls displayed normal myelin integrity, but from 7 days to 1 month after BCAS, there were indications of a time‐dependent increase in severity index (Figure S1F–I). Overall, these findings (Figure S1) demonstrate that following BCAS, mice with chronic cerebral hypoperfusion showed a considerable disturbance of white matter and demyelination, and that the crucial time point for histological evidence of white matter abnormalities and demyelination was at 14 days' post‐injury.

### 3.2 EAAT3 inhibition promotes remyelination for white matter repair

**FIGURE 2:**
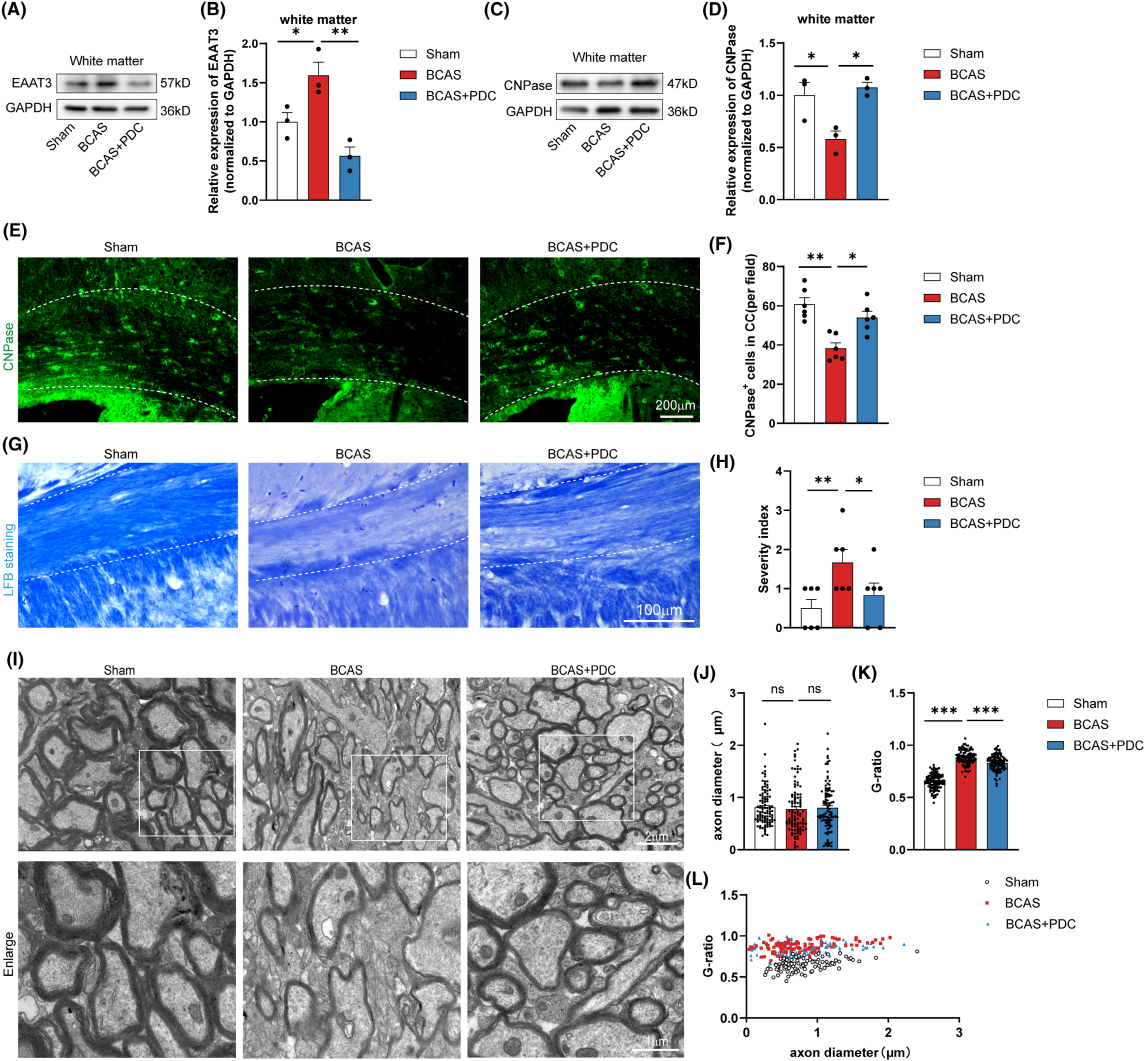
White matter damage and demyelination in BCAS mice with chronic cerebral hypoperfusion can be rescued by injection of PDC. (A, B) Representative immunoblotting and quantification showed that the expression of EAAT3 decreased after injection of the inhibitor. The GAPDH protein served as a control. (C, D) Representative immunoblotting and quantification showed that the expression of CNPase can increase after injection of PDC. The GAPDH protein served as a control. (E) Representative images of CNPase (green) in CC of each group. (F) Results of quantitative analysis of the CNPase‐positive cells in each field of CC. *n* = 3 in each group, 2 or 3 images per animal. (G, H) Representative LFB‐stained images and quantification illustrating preserved white matter integrity due to decreased myelin rarefaction and white matter lesion formation in the corpus callosum (medial) on 14 days in PDC mice compared with the BCAS group. *n* = 3 in each group, 2 or 3 images per animal. (I–K) Representative electron microscopy images and quantification of axon diameter and G‐ratio on 14 days in indicated groups. (L) G‐ratio plotted as a function of axon diameter, *n* = 3 in each group. There was no difference in body weight between mice in each group. The data for H dose not exhibit a normal distribution. *p* Value was determined by ANOVA with the Kruskal–Wallis test. In addition, the data conformed to a normal distribution. *p* Value was determined by ANOVA with Bonferroni's post‐hoc test. *p < 0.05, **p < 0.01, between the indicated groups, ns indicates non‐significance, *n* = 3 in each group. Data are represented as means ± SEM.

To clarify the possible role of oligodendroglial EAAT3 in the pathological progress of white matter damage following chronic cerebral hypoperfusion, PDC, a potent and selective EAAT3 inhibitor was used. Western blot results revealed that EAAT3 expression in white matter increased dramatically 14 days after BCAS, as expect, the continuous intracerebroventricular administration of PDC significantly reversed this upregulation of EAAT3 expression (Figure 2A,B). Interestingly, a quantitative analysis of CNPase expression in BCAS mice's white matter by western blot showed markedly reduction of CNPase expression compared with controls on Day 14, while a significantly upregulation of CNPase expression after PDC treatment was observed in the BCAS mice (Figure 2C,D). An analysis of CNPase expression by immunostaining showed consistent results with the western blot experiments (Figure 2E,F).

To further examine whether inhibition of EAAT3 contributed to axonal remyelination after white matter injury, myelin loss of the corpus callosum was evaluated by LFB staining. The percentage of myelin loss in mice exposed to BCAS increased compared to a sham group on Day 14, whereas PDC administrated after BCAS reversed this increase (Figure 2G,H). The myelin sheaths in PDC‐treated BCAS mice were thinner (i.e., had a lower G‐ratio, or the ratio between the inner and outer diameter of the myelin sheath) than those in BCAS mice, according to scanning electron microscopy (Figure 2I,K,L), even though the diameter of the myelinated axons in the corpus callosum did not vary between PDC‐treated BCAS mice and BCAS mice (Figure 2J).

### 3.3 EAAT3 inhibition promotes OPCs' proliferation and migration for remyelination

**FIGURE 3:**
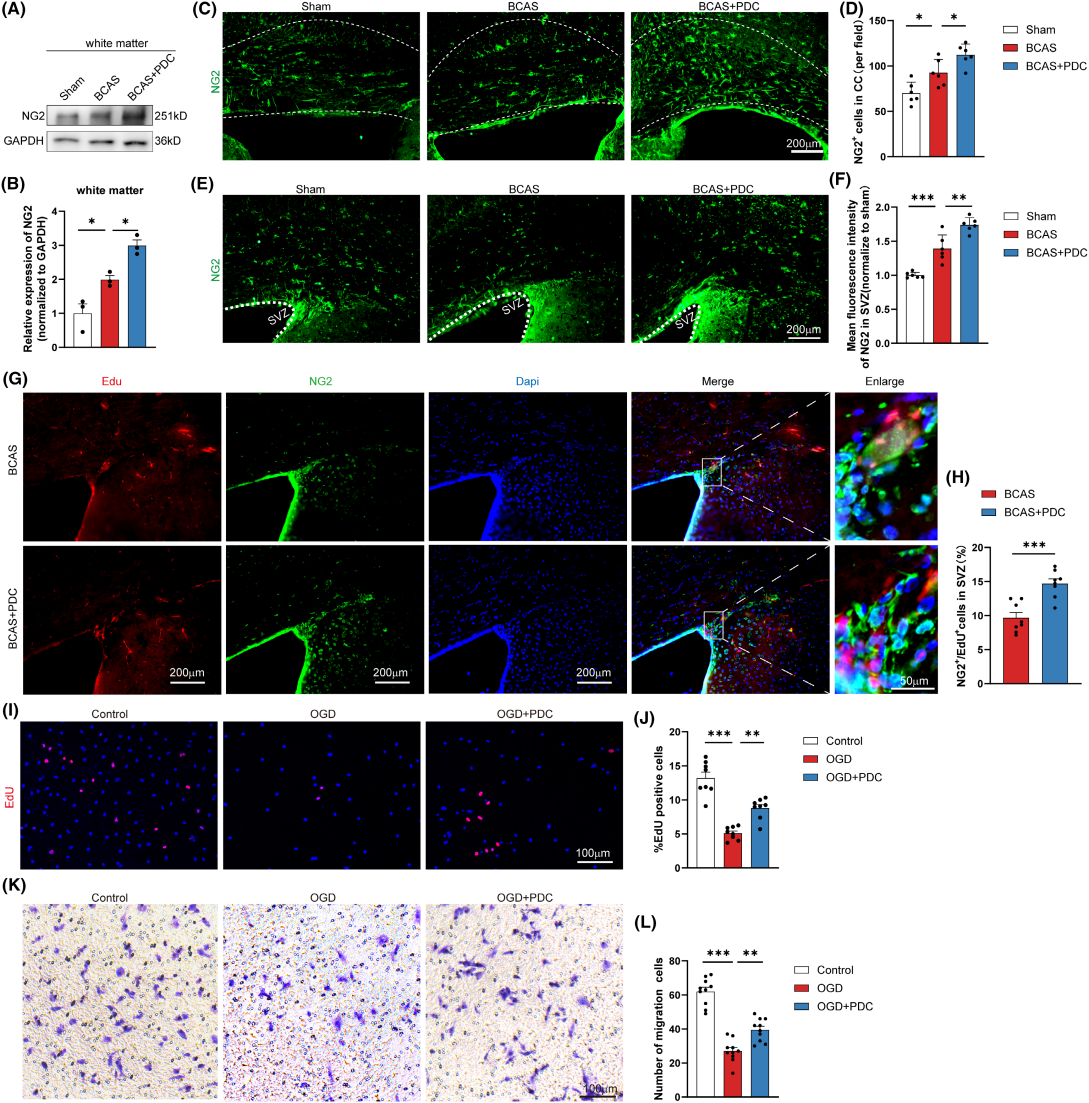
PDC administration increases the survival, proliferation, and migration of OPCs. (A, B) Representative immunoblotting and quantification showed the expression of NG2 in white matter on Day 14 after BCAS and the expression can increase after injection of PDC. (C) Representative images of NG2 expression in CC in each group. (D) Results of quantitative analysis of the NG2‐positive cells in each field of CC in each group, *n* = 3, 2 or 3 images per animal. (E) Representative images of NG2 expression in SVZ in each group. (F) Results of immunofluorescent intensity of the NG2‐positive cells in each field of SVZ in each group, *n* = 3, 2 or 3 images per animal. (G) Representative immunofluorescence co‐staining images of EdU (red) and NG2 (green) in SVZ in each group. (H) Quantitative analysis of % EdU^+^/ NG2^+^ cells in total DAPI of SVZ, *n* = 3, 2 or 3 images per animal. (I) Representative immunofluorescence images of EdU immunoreactive primary OPCs in the indicated groups. (J) % EdU‐positive OPCs in total DAPI in the indicated groups, *n* = 3–5 images from at least three independent experiments. (K) Representative photomicrographs of migrated OPCs. (L) Quantitative analysis of the numbers of migrated OPCs, *n* = 3–5 images from at least three independent experiments. There was no difference in body weight between mice in each group. The data for each group conformed to a normal distribution. *p* Value was determined by ANOVA with Bonferroni's post‐hoc test. The data of H were analyzed using the Mann–Whitney *U* test. *p < 0.05, **p < 0.01, between the indicated groups, ns indicates non‐significance. Data are represented as means ± SEM.

The majority of the available data points to OPCs as the source of remyelinating oligodendrocytes, thus we next investigated whether OPCs influenced the way that EAAT3 inhibition promoted remyelination. We firstly assessed the status of OPCs in corpus callosum at 1 day, 7 days, 14 days, and 1‐month post‐BCAS. We found that the density of OPCs (NG2^+^ cells) in the corpus callosum persistently increased, and it peaking at 14 days post‐BCAS (Figure S2A,B). Kinetically, NG2 expression indicated by western blot also showed an increasing trend, and on Day 14, a significantly increase was observed compared with the sham group (Figure S2C,D). Subsequently, we evaluated the effect of EAAT3 inhibition on OPCs. We found a further significant elaboration of NG2 protein expression at 14 days post‐BCAS after PDC treatment (Figure 3A,B). In addition, there was a significant enlargement in the density of OPCs (NG2^+^ cells) in both the corpus callosum and subventricular zone at 14 d after PDC treatment in BCAS mice (Figure 3C–F). Previous research indicated that the SVZ contains a sizable pool of OPCs. Following injury, these SVZ‐derived OPCs are successfully recruited to the corpus callosum to replace harmed oligodendrocytes. We then evaluated the proliferation and migration of OPCs in vivo and in vitro. Based on the EdU incorporation experiment, OPCs multiplied more quickly in the PDC‐treated brains than in the BCAS mice brain cells (Figure 3G,H). Additionally, in vitro, PDC treatment dramatically increased the percentage of EdU‐positive OPCs compared to OGD cells (Figure 3I,J). Using transwell migration assays, we then evaluated the migration of OPCs. When compared to OGD‐treated OPCs, PDC treatment sped up OPC migration (Figure 3K,L).

### 3.4 EAAT3 inhibition promotes OPCs differentiation for remyelination

**FIGURE 4:**
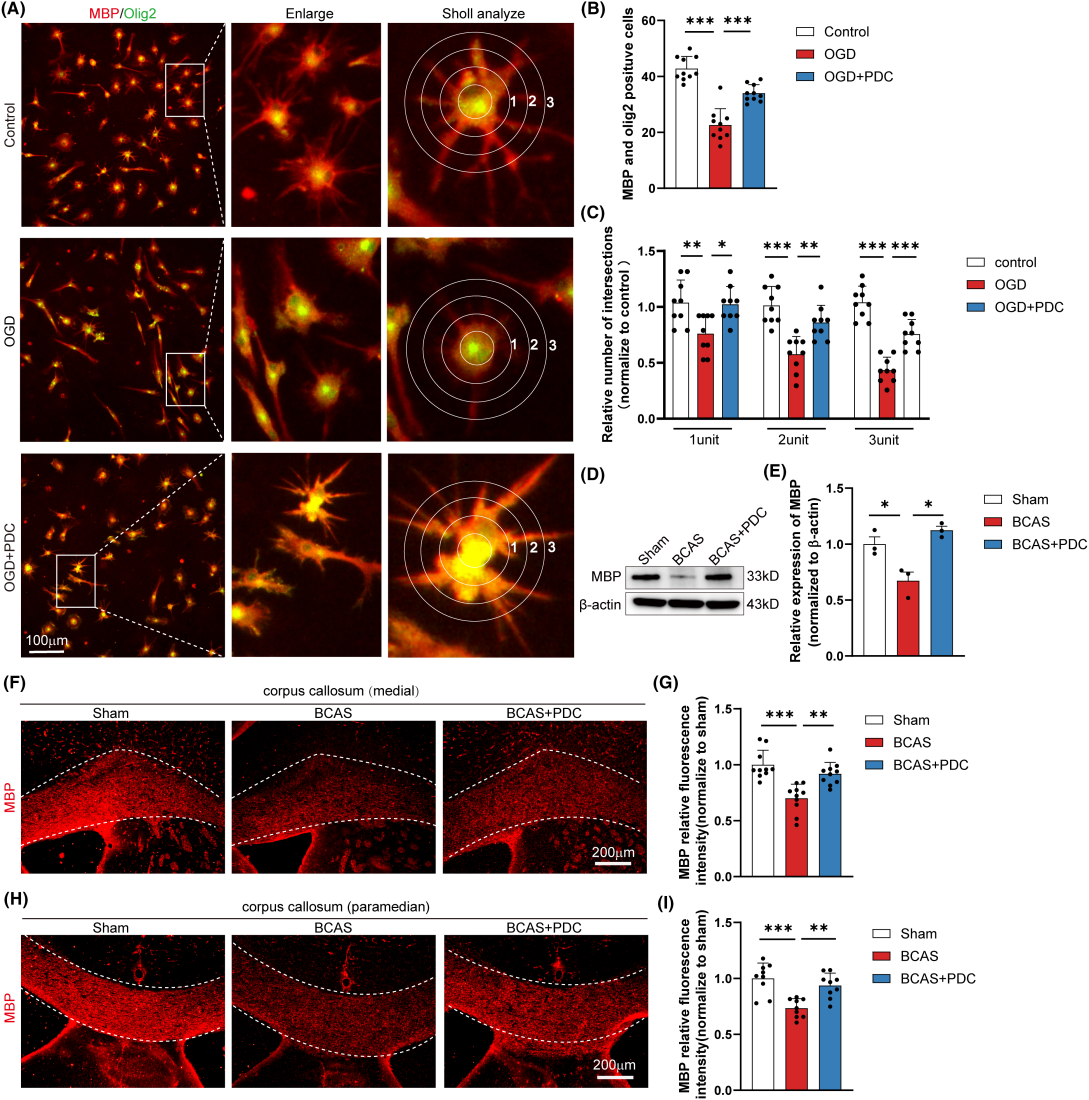
PDC administration increases the differentiation of OPCs. (A) Representative immunofluorescence co‐staining images of MBP (red) and Olig2 (green) in primary oligodendrocytes. (B) Quantitative analysis of the number of MBP^+^/ Olig2^+^ cells in each field of the indicated groups. *n* = 3–5 images from at least three independent experiments. (C) The process extensions for primary oligodendrocytes were analyzed using Sholl analysis. By counting the intersections that processes make with concentric circles that are numbered 1–3 to represent increasing distance from the cell body, branching was measured, *n* = 3–5 images from at least three independent experiments. (D, E) Representative immunoblotting and quantification of MBP in each group, *n* = 3 mice. (F–I) Representative immunofluorescence images and quantification of MBP immunofluorescent intensity in media and paramedian of CC in each field of the indicated groups, *n* = 3, 3–5 images per animal. There was no difference in body weight between mice in each group. The data for each group conformed to a normal distribution. *p* Value was determined by ANOVA with Bonferroni's post‐hoc test. *p < 0.05, **P < 0.01, between the indicated groups, and ns indicates non‐significance. Data are represented as means ± SEM.

The biological process of recapitulating myelination known as remyelination necessitates the differentiation of OPCs into mature oligodendrocytes. Oligodendrocytes at various stages were co‐stained with immunocytochemistry to see whether EAAT3 also regulates OPCs differentiation. When compared to cells treated with OGD, we discovered that PDC treatment dramatically enhanced the number of MBP^+^/Olig2^+^ primary cells (Figure 4A,B). Sholl analysis was also used to measure the morphological complexity of the differentiating OPCs processes because oligodendrocytes change during differentiation from having a straightforward bipolar morphology to having a complicated branched morphology. In comparison with OGD‐treated cells, the results showed that PDC‐treated primary cells considerably increased the number of intersections made by the outer circle with the longer processes (three units), indicating promotion of morphological differentiation (Figure 4A,C). Similar to this, western blot research (Figure 4D,E) reveals that PDC therapy in BCAS mice dramatically raised the expression of the mature oligodendrocyte marker (MBP). Meanwhile, 14 days after BCAS, MBP expression in the medial and paramedian zones of the CC substantially decreased, according to immunostaining studies. However, intracerebroventricular injection of PDC after BCAS reversed this downregulation of MBP expression (Figure 4F–I).

**FIGURE 5:**
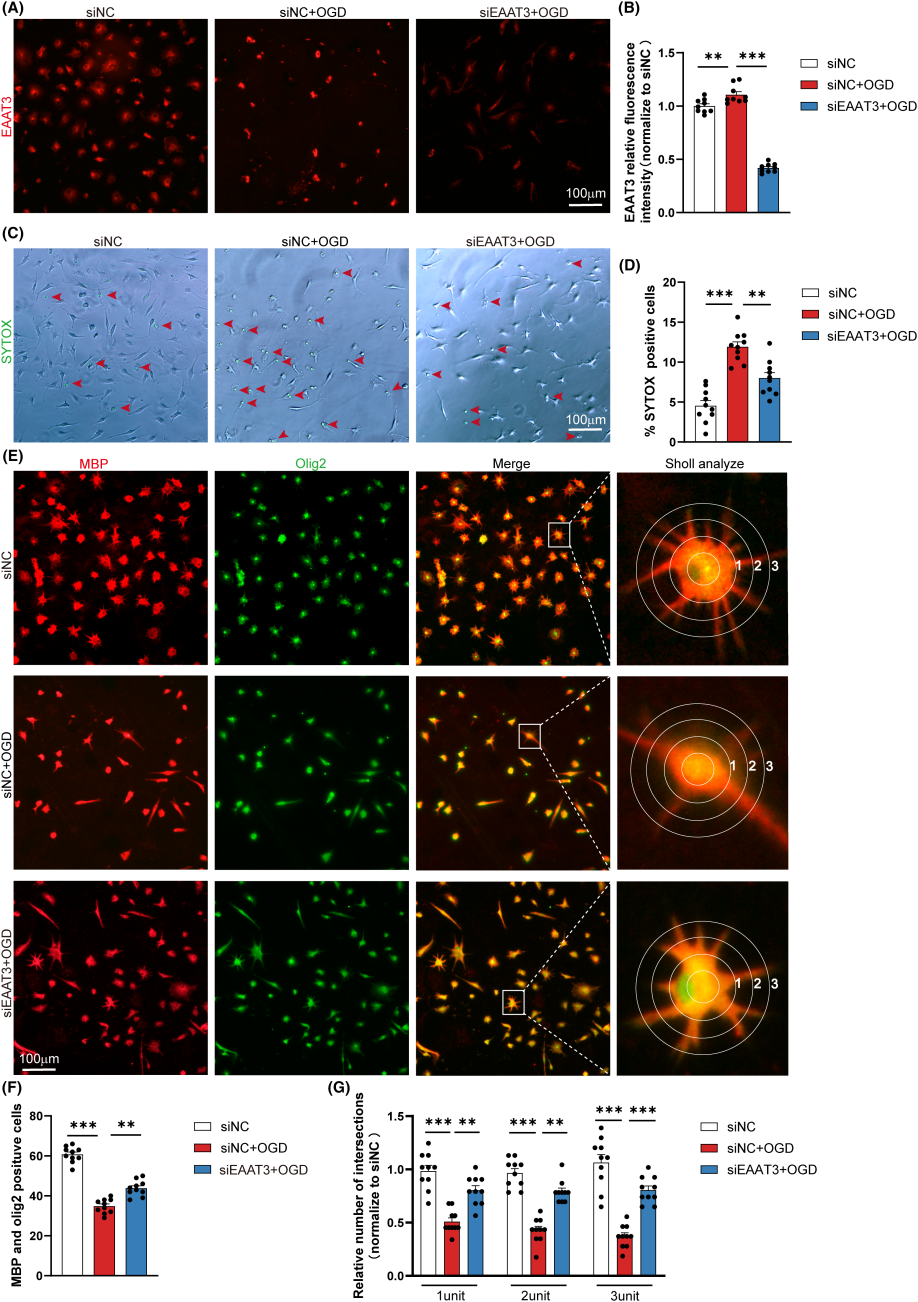
EAAT3 knockdown motivates OPCs differentiation for remyelination. (A) Representative immunofluorescence images of EAAT3 (red) after transfection. (B) Mean fluorescence intensity quantitative analysis of EAAT3 in each field of the indicated groups. *n* = 3–5 images from at least three independent experiments. (C) Representative images and quantitative analysis (D) of the percentage of SYTOX‐positive cells in all cells under each field, *n* = 3–5 images from at least three independent experiments. (E) Representative immunofluorescence co‐staining images of MBP (red) and Olig2 (green) in oligodendrocytes. (F) Quantitative analysis of the number of MBP^+^/Olig2^+^ cells in each field of the indicated groups. *n* = 3–5 images from at least three independent experiments. (G) The process extensions for primary oligodendrocytes were analyzed using Sholl analysis. By counting the intersections that processes make with concentric circles that are numbered 1–3 to represent increasing distance from the cell body, branching was measured. *n* = 3–5 images from at least three independent experiments. The data for each group conformed to a normal distribution. *p* Value was determined by ANOVA with Bonferroni's post‐hoc test. *p < 0.05, **p < 0.01, between the indicated groups, and ns indicates non‐significance. Data are represented as means ± SEM.

To investigate the effect of cell‐specific EAAT3 inhibition further, genetic knockdown of EAAT3 with siRNA was employed in OPCs. In line with our findings in PDC, EAAT3 expression was considerably elevated in OPCs after OGD therapy. Furthermore, siRNA‐mediated EAAT3 knockdown significantly reduced OGD‐induced EAAT3 expression in OPCs (Figure 5A,B). To investigate the role of EAAT3 silencing in OGD‐induced OPCs damage further, cell viability was measured using the SYTOX nucleic acid stain. Cell viability in OPCs was considerably reduced under OGD conditions, as demonstrated in Figure 5C,D. When compared to scramble vector‐transfected cells, EAAT3 silencing significantly enhanced cell viability after OGD therapy. Finally, similar to the PDC treatment results, we noticed that silencing EAAT3 significantly increased the number of MBP^+^/Olig2^+^ primary cells (Figure 5E,F).

### 3.5 mTOR signaling pathway is contributing to EAAT3 inhibition–induction remyelination

**FIGURE 6:**
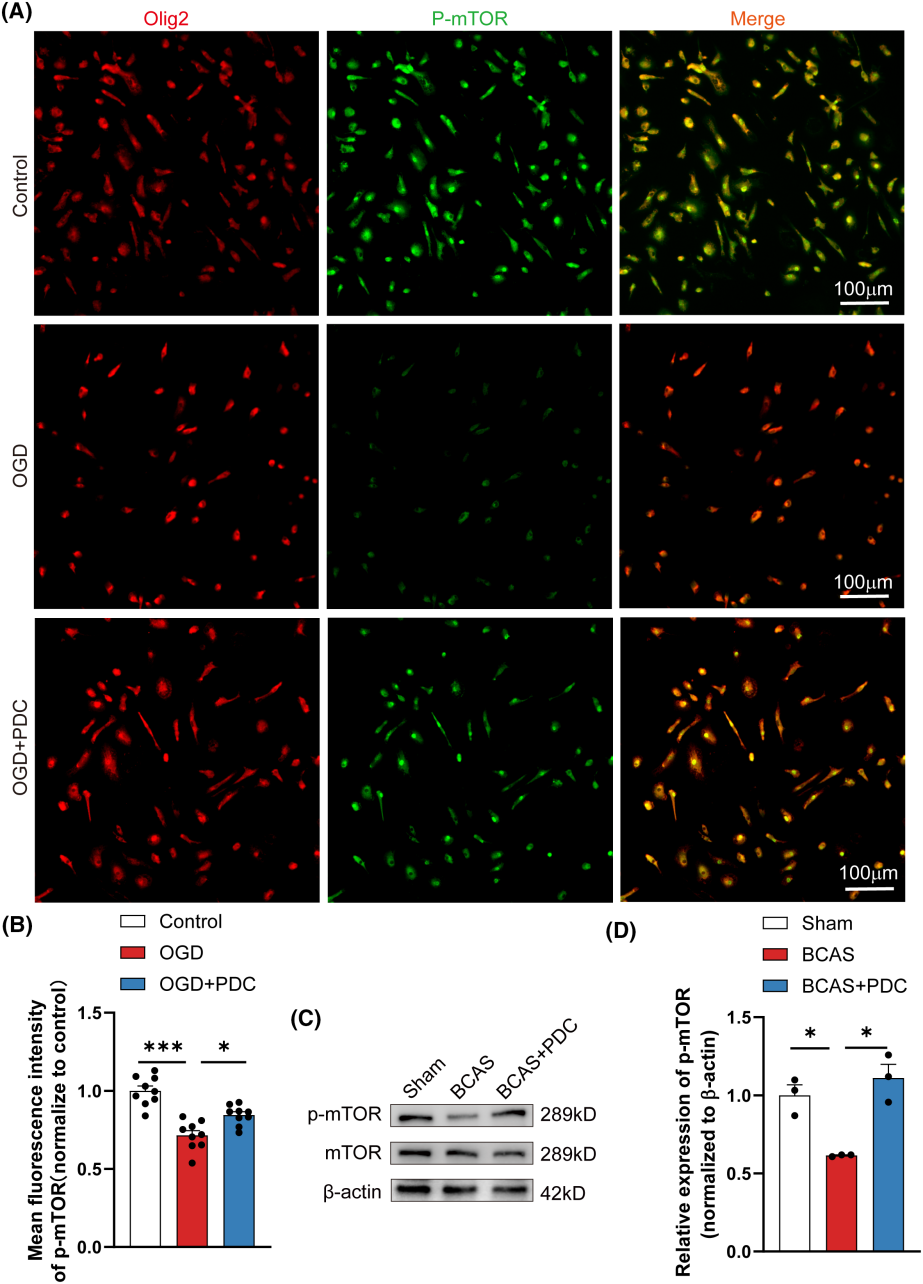
PDC administration activates the mTOR signaling pathway in vitro. (A) Representative immunofluorescence co‐staining images of Olig2 (red) and P‐mTOR (green) in primary OPCs. (B) Mean fluorescence intensity quantitative analysis of P‐mTOR in each field of the indicated groups. *n* = 3–5 images from at least three independent experiments. (C, D) Representative immunoblotting and quantification of P‐mTOR in indicated groups, *n* = 3 mice. There was no difference in body weight between mice in each group. The data for each group conformed to a normal distribution. *p* Value was determined by ANOVA with Bonferroni's post‐hoc test. *p < 0.05, **p < 0.01, between the indicated groups, ns indicates non‐significance. Data are represented as means ± SEM.

It was not obvious how EAAT3 inhibition controlled remyelination in response to white matter damage in the studies above. Since the mTOR signaling pathway is crucial for controlling cell growth, differentiation, and migration, it was investigated. As a result, we looked at alterations in oligodendrocyte P‐mTOR activation.^36^ The expression of P‐mTOR was lower in the OGD group compared to the control group, according to double immunofluorescence labeling. The expression of P‐mTOR, in contrast, was considerably higher in the PDC group following treatment than in the OGD group (Figure 6A,B). Correspondingly, western blot analysis revealed that the expression of P‐mTOR was considerably downregulated in the OGD group compared to the control group, which was corrected following PDC therapy (Figure 6C,D).

**FIGURE 7:**
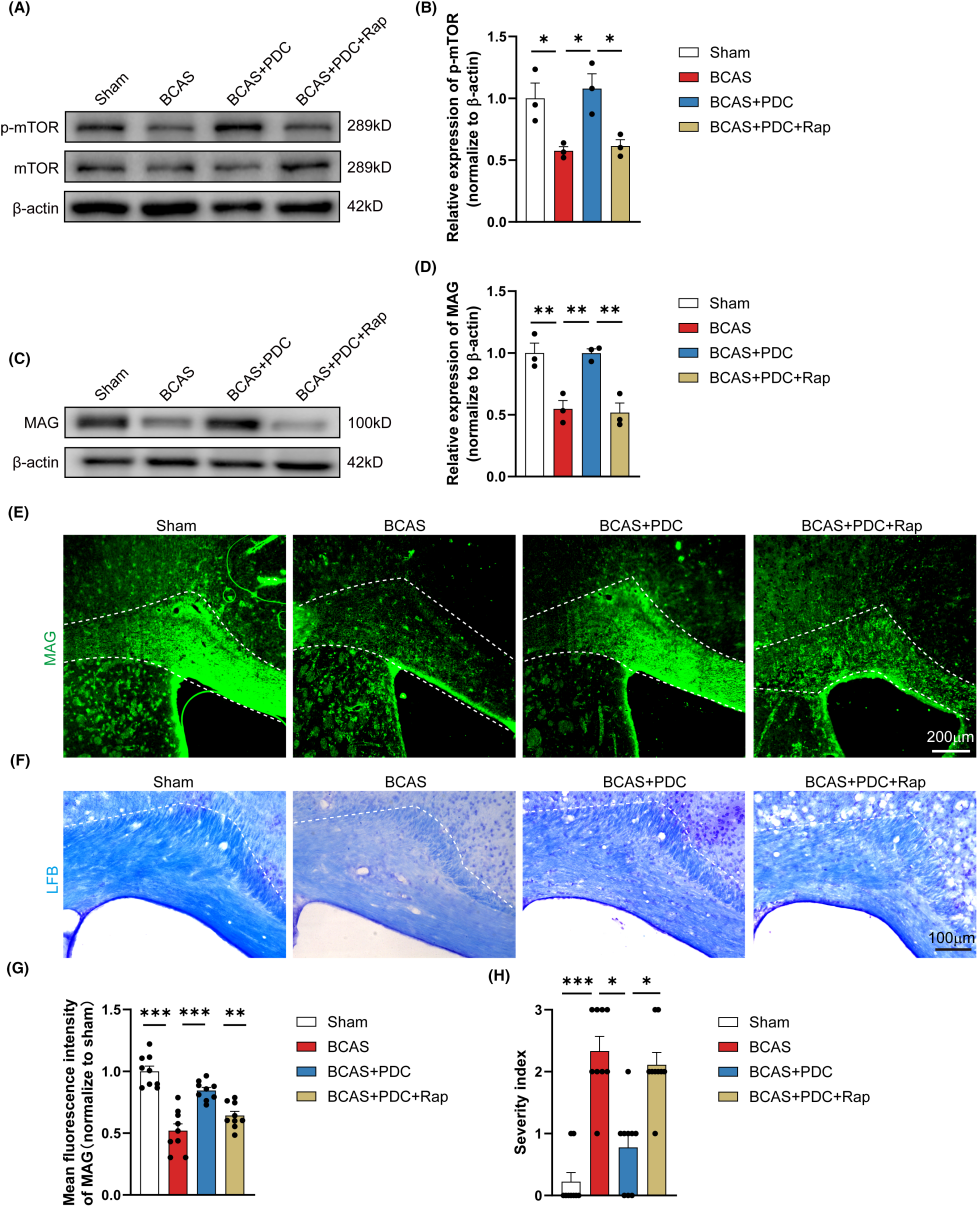
Inhibition of the mTOR signaling pathway can hinder the protective effect of PDC. (A, B) Representative immunoblotting and quantification of P‐mTOR and mTOR in indicated groups. The β‐Actin protein served as a control, *n* = 3 mice. (C, D) Representative immunoblotting and quantification of MAG in indicated groups. The β‐Actin protein served as a control, *n* = 3 mice. (E, G) Representative immunofluorescence images and quantification of MAG immunofluorescent intensity of CC in each field of the indicated groups. *n* = 3, 3–5 images per animal. (F, H) Representative LFB‐stained images and quantification illustrating preserved white matter integrity due to decreased myelin rarefaction and white matter lesion formation in CC in each group. *n* = 3, 3–5 images per animal. There was no difference in body weight between mice in each group. The data for H that do not exhibit a normal distribution. *p* Value was determined by ANOVA with the Kruskal–Wallis test. In addition, the data conformed to a normal distribution. *p* Value was determined by ANOVA with Bonferroni's post‐hoc test. *p < 0.05, **p < 0.01, between the indicated groups, ns indicates non‐significance. Data are represented as means ± SEM.

The next step was to see whether the mTOR signaling pathway directly influenced EAAT3 remyelination after white matter injury. When compared to PDC therapy alone, mTOR inhibition with a specific antagonist, rapamycin (Rap), resulted in significantly lower P‐mTOR levels (Figure 7A,B). As a result, we investigated the effect of inhibiting the mTOR signaling pathway on remyelination. In western blot experiments, the protein level of MAG, a mature oligodendrocyte marker, was significantly higher in the PDC‐treated group than in the BCAS model group. It was, however, dramatically reduced after combining Rap with PDC therapy in BCAS mice (Figure 7C,D). Similarly, we confirmed the similar effects in MAG immunofluorescence assays (Figure 7E,G). LFB staining was also used to detect myelin degeneration in the corpus callosum. PDC therapy was shown to minimize the percentage of myelin loss in mice when compared to the model group. However, combining Rap with PDC therapy significantly reversed it (Figure 7F,H).

### 3.6 EAAT3 inhibition promotes OPCs survival and improves neurologic function recovery of mice after white matter injury

**FIGURE 8:**
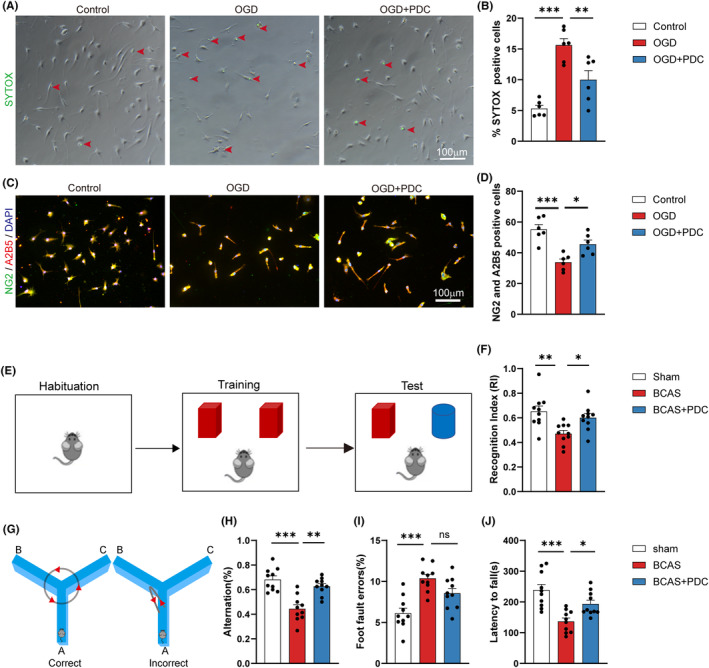
Inhibition of EAAT3 enhances primary OPCs survival and promotes the behavioral defects of BCAS mice. (A) Representative images and (B) quantitative analysis of the percentage of SYTOX‐positive cells in all cells under each field, *n* = 3–5 images from at least three independent experiments. (C) Representative immunofluorescence co‐staining images of A2B5 (red), NG2 (green), and DAPI (blue) in primary OPCs. (D) Quantitative analysis of A2B5^+^/ NG2^+^ cells in each field, *n* = 3–5 images from at least three independent experiments. (E, F) The pattern diagram and the exploratory preference for novel objects in the novel object recognition results 1‐month after BCAS, *n* = 10 mice. (G, H) The percentage of spontaneous alternations in the Y‐maze test results 1‐month after BCAS, *n* = 10 mice. (I) Grid walking test results 1‐month after BCAS, *n* = 10 mice. (J) Rotarod test results 1‐month after BCAS, *n* = 10 mice. There was no difference in body weight between mice in each group. The data for each group conformed to a normal distribution. *p* Value was determined by ANOVA with Bonferroni's post‐hoc test. *p < 0.05, **p < 0.01, between the indicated groups, and ns indicates non‐significance. Data are represented as means ± SEM.

Next, we looked deeper into whether EAAT3 inhibition had a positive impact on OPCs survival and neurological function following white matter damage. First, we use the SYTOX Green nucleic acid stain to determine the impact of EAAT3 inhibition on OPCs survival. Cell viability was dramatically reduced under the OGD condition, as seen in Figure 8A,B. On the other hand, PDC improved oligodendrocyte viability following OGD. The quantity of OPCs was then investigated using OPC markers. We saw that there were much less OPCs than there would have been under normal circumstances. As opposed to OGD‐treated cells, the presence of PDC exposure dramatically increased the amount of OPCs (Figure 8C,D). Additionally, a number of behavioral tests were used to assess the recovery of mice's neurologic function following a white matter lesion. White matter lesions typically impair cognition, balance, and movement. At 1 month following BCAS, mice were first put through a series of cognitive behavior tests, including the Y‐maze test and object identification tests, which look at spatial and non‐spatial working memory, respectively. In the object recognition tests, the BCAS mice showed a substantial decline in their capacity to discriminate, whereas the PDC mice showed a significant increase in their ability to discriminate (Figure 8E,F). BCAS animals had a lower rate of spontaneous alternations in the Y‐maze tests than sham‐operated mice did. After a month of BCAS, PDC therapy, however, could increase the frequency of spontaneous alternations (Figure 8G,H). Second, earlier research revealed that the mice with BCAS were impaired in their ability to move and use their muscles. So, to assess the mice's ability to move about and their motor skills, we utilized the foot‐fault test and the rotarod test. At 1 month following BCAS, mice showed severe behavioral abnormalities of the left forepaw as measured by the foot‐fault test for putting and grabbing. Interestingly, mice had a propensity for considerable recovery at 1 month after receiving BCAS with PDC therapy (Figure 8I). In rotarod experiments, BCAS mice displayed considerably shorter times spent maintaining balance. The test performance in PDC‐treated mice significantly improved (Figure 8J).

Previous reports indicated that CBF in the brain surface had a substantial reduction in BCAS mice.^37^ Laser speckle contrast imaging provides a rapid characterization of cortical flow dynamics for functional monitoring of the microcirculation. Therefore, laser speckle images of temporal changes in CBF responses to PDC treatment at the pre‐operation, after operation, 1 day, 7 days, 14 days, and 1‐month post‐injury time points were evaluated (Figure S3A). CBF did not relevantly influence at each individual observation time point in sham‐treated mice. Compared with the sham‐operated mice, the CBF values (ratio to the preoperative baseline) in the BCAS group decreased significantly after operation (*p* < 0.05). At Day 1, the CBF values began to recover but remained significantly lower in comparison with the sham group (*p* < 0.05). We then focused on the cortical perfusion response after PDC. PDC‐treated BCAS mice did at first not differ from BCAS group but remained ~50% below the physiological blood flow response of sham‐treated animals at after operation and Day 1. By Day 7, we observed a slightly higher rate of cerebral blood flow recovery in PDC‐treated animals compared with BCAS animals (Figure S3B).

## 4 DISCUSSION

EAAT3, also known as glutamate transporter 1, is an important protein that functions to regulate the levels of glutamate in the central nervous system.^38^, ^39^ Several studies have demonstrated that dysregulation of EAAT3 expression or function can lead to various neurological disorders, including Alzheimer's disease, Parkinson's disease, depression, and schizophrenia.^40^, ^41^ For example, reduced expression of EAAT3 has been observed in the prefrontal cortex of subjects with schizophrenia, and this reduction is thought to contribute to increased glutamate levels and NMDA receptor hyperactivity, which may underlie the cognitive and behavioral impairments associated with this disorder.^42^ However, little is known about how EAAT3 affects neurological disorders involving anomalies in the white matter, such as demyelinating illnesses. In this research, we showed for the first time that abnormal EAAT3 is strongly high expressed after white matter relative demyelination (Figure 1) and that suppressing EAAT3 through antagonist PDC administration significantly enhances remyelination, alleviates cognitive and motor deficits, and promotes cerebral blood flow recovery (Figures 2 and 8; Figure S3), suggesting that EAAT3 plays a deleterious function in the white matter repair. Notably, when we looked at the expression levels of EAAT3 during brain development, the findings revealed that EAAT3 did not vary considerably at a number of critical times during the process (Figure 1E–H). Additionally, we used traumatic brain injury and intracerebral hemorrhage models to imitate acute brain injury. The results likewise demonstrated that there was no discernible difference in the expression level of EAAT3 following acute brain injury (Figure 1I–K). Besides that, in these tests, neuronal enrichment areas are represented by the hippocampus and cortex, and oligodendrocyte enrichment areas are represented by white matter. The foregoing results demonstrated that EAAT3 did not change considerably at the levels of neurons and oligodendrocytes at different stages of brain development, and the same did not change significantly at the levels of neurons and oligodendrocytes following acute brain damage (Figure 1E–K). However, it should be emphasized that there are still variances in the expression levels of individual mice in our stated experiments, and so our experiments cannot totally rule out a role for EAAT3 in the described experimental model. In contrast, only in BCAS‐induced chronic white matter injury did EAAT3 alterations in neurons become significantly different from those in oligodendrocytes, further supporting the idea that EAAT3 has a unique role in oligodendrocytes in white matter lesions (Figure 1L–O). In addition, the white matter is particularly vulnerable to changes in CBF due to its high metabolic demand and reliance on a constant supply of oxygen and nutrients. Low CBF is linked to the severity of white matter hyperintensities on T2‐weighted MRI, according to cross‐sectional studies. In the present study, we conducted experiments using a mouse model of chronic cerebral hypoperfusion through BCAS surgery with microcoils. It duplicates the white matter injuries and associated behavior deficits, causing a notable decreased in CBF and a lack of axonal myelination in the corpus callosum (CC) and gradual demyelination (Figure S1). Because of this, EAAT3 might be thought of as a therapeutic target for neurological conditions involving white matter diseases, and its inhibitors may be useful in treating these situations.

When white matter relative demyelination occurs, oligodendrocytes can become damaged or lost, leading to a decrease in myelin production and an impairment of nerve function.^43^ In response to demyelination, OPCs can differentiate into mature oligodendrocytes and remyelinate the denuded axons.^44^ OPCs are numerous and constantly poised to multiply to replace oligodendrocytes and myelin in the adult central nervous system. Previous research demonstrated that OPCs mobilized after BCAS and had increased proliferative potential.^45^ However, OPCs' proliferation was gradually stopped under persistent hypoxia. As a result, BCAS‐induced demyelination was continuously sustained and enhanced due to the effects on OPCs mobilization and significant oligodendrocyte mortality. Additionally, research on postmortem human brains have revealed that while the number of mature oligodendrocytes declines in ischemic white matter lesions in patients, the number of OPCs increases, indicating that recruitment or differentiation is suppressed following chronic cerebral hypoperfusion.^46^ As a result, influencing OPC cell proliferation, migration, and differentiation may noticeably facilitate the treatment of white matter injury. Interesting, in the present study, single‐cell RNA sequencing data showed that EAAT3 was highly expressed in OPCs and mature oligodendrocytes as compared with other neural cells (Figure 1A–C). However, the role of EAAT3 in oligodendroglial cells has never been established. Based on our current findings, we believe EAAT3 may promote white matter repair by modulating OPCs. The main sources of OPCs for remyelination are the SVZ and the CC. We further confirmed that inhibiting EAAT3 improves OPCs survival in the CC and SVZ zone (Figure S2; Figure 3A–F), promotes SVZ‐derived OPCs proliferation (Figure 3G,H), and stimulates OPCs proliferation (Figure 3I,J), migration (Figure 3K,L), and differentiation in vitro (Figures 4 and 5). Because remyelination can be caused by the proliferation and recruitment of freshly produced OPCs to demyelinating lesions, followed by differentiation into myelin, our findings indicate that inhibiting EAAT3 in OPCs resulted in the restoration of myelination in the CC and, ultimately, white matter repair. More importantly, the identification of inhibitory variables that operate on OPC functions may pave the way for endogenous regenerative progress toward functional recovery in the treatment of demyelinating disorders. These findings also revealed that inhibiting EAAT3 in OPCs resulted in the restoration of myelination in the CC, which could eventually lead to white matter repair.

The mechanistic target of rapamycin (mTOR) pathway is a conserved cellular signaling pathway that regulates various cellular processes such as growth, migration, and metabolism.^47^, ^48^ Recent studies have shown that mTOR signaling can modulate EAAT3 activity by phosphorylating protein kinase B, which in turn phosphorylates and activates the surface expression of EAAT3.^49^ This mechanism of action has been shown to be critical in regulating synaptic transmission and preventing excitotoxicity. However, research also found that mTOR depletion affected OPCs' genesis and proliferation, as well as myelination in the CC during early postnatal phases.^50^, ^51^ As a result, we hypothesize that EAAT3 modulation of the mTOR signaling pathway may also repair white matter injury by influencing the involvement of OPCs. As expected, we discovered that aberrant EAAT3 blocked the mTOR pathway, which was important for OPC proliferation and differentiation following white matter injury (Figures 6 and 7).

To summarize the results of our experiments, we also found that in vivo, suppression of EAAT3 levels by an EAAT3‐specific antagonist restored demyelination damage, whereas in vitro, direct knockdown of EAAT3 greatly enhanced the development and maturation of OPCs. Furthermore, we established in vivo and in vitro that EAAT3 affects demyelination through blocking the mTOR signaling pathway. These findings largely show that EAAT3 has a direct influence on demyelination.

There are various limitations to our study. To begin, we have not confirmed the role of EAAT in white matter repair in EAAT3 knockout mice. Second, the mechanism underlying EAA3 increase following chronic cerebral hypoperfusion is unknown. Furthermore, because the antagonist PDC used in this study has been shown to inhibit both EAAT1 and EAAT3, an indirect action of EAAT1 on white matter repair cannot be completely ruled out; furthermore, research is needed. To address this problem, future research would look into the role of cellular specific EAAT3 using conditional knockout mice. Furthermore, no research has been reported on the expression and distribution of oligodendrocyte EAAT3 in individuals with white matter injury‐related disorders. We only noted one report of EAAT3 expression levels using qPCR in 20 INF‐β‐treated multiple sclerosis patients. The results demonstrated that EAAT3 expression was significantly decreased at 3 months of treatment compared to the baseline, indicating that a higher EAAT3 expression in multiple sclerosis patients is deleterious.^52^ In addition, human pathology samples, particularly brain tissue, are more difficult to obtain. It may be possible to perform research using imaging data, blood, and metabolite samples. Furthermore, clinical investigations of antagonists targeting EAAT3 targets should be expanded. As a result, our findings are still far from therapeutic application and must be investigated further in clinical samples.

Taken together, although EAAT3, an excitatory amino acid transporter, is essential for maintaining neurotransmission and guarding against excitotoxicity, the function of oligodendroglial EAAT3 has never been described. Our research has shown that EAAT3 negatively regulates OPCs' proliferation, migration, and differentiation. These OPCs' benefits from EAAT3 suppression facilitate oligodendroglial survival, white matter remyelination, and ultimately functional recovery. EAAT3 inhibition mediates remyelination and white matter repair through the phosphorylated mTOR pathway. These findings imply that EAAT3‐targeting approaches may have therapeutic benefits in the management of demyelinating diseases or other neurological conditions involving white matter abnormalities, but further research including using conditional knockout mice is required to clarify the intricate interplay between the role of oligodendroglial‐specific EAAT3 in white matter repair and remyelinating function.

## AUTHOR CONTRIBUTIONS

Yingmei Zhang performed the experiments, analyzed the data, and wrote the paper. Dongshan Ya, Jiaxin Yang, and Xiaoxia Li contributed to some parts of the experiments. Yanlin Jiang, Ning Tian, and Jiawen Wang designed some parts of the experiments. Jungang Deng, Bin Yang, and Qinghua Li provided financial support and advice on the interpretation of the data. Rujia Liao conceived of and designed the experiment, analyzed and interpreted the data, provided financial support, and wrote the study. All authors have read and agreed to the published version of the manuscript.

## CONFLICT OF INTEREST STATEMENT

The authors declare that they have no known competing financial interests or personal relationships that could have appeared to influence the work reported in this study.

## Supporting information


Figure S1

Figure S2

Figure S3

Figure S4

## Data Availability

The datasets used and/or analyzed during the current study are available from the corresponding author on reasonable request.
