# Supplementary figures and images for: EAAT3 impedes oligodendrocyte remyelination in chronic cerebral hypoperfusion‐induced white matter injury
Source: CNS Neurosci Ther. 2023 Oct 6;30(1):e14487. doi: 10.1111/cns.14487 (PMC10805396; doi:10.1111/cns.14487)

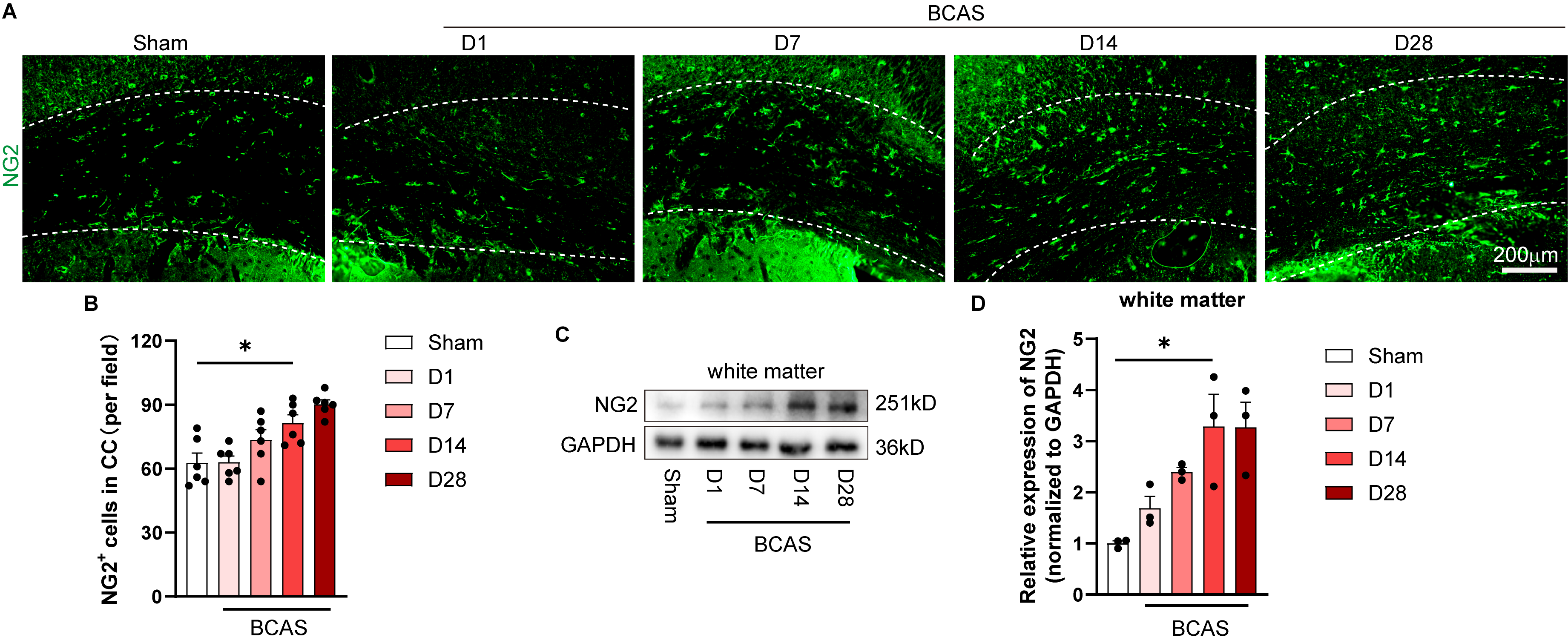

Supplement: Supplementary file 2 — Figure S2 [file CNS-30-e14487-s001.tif]

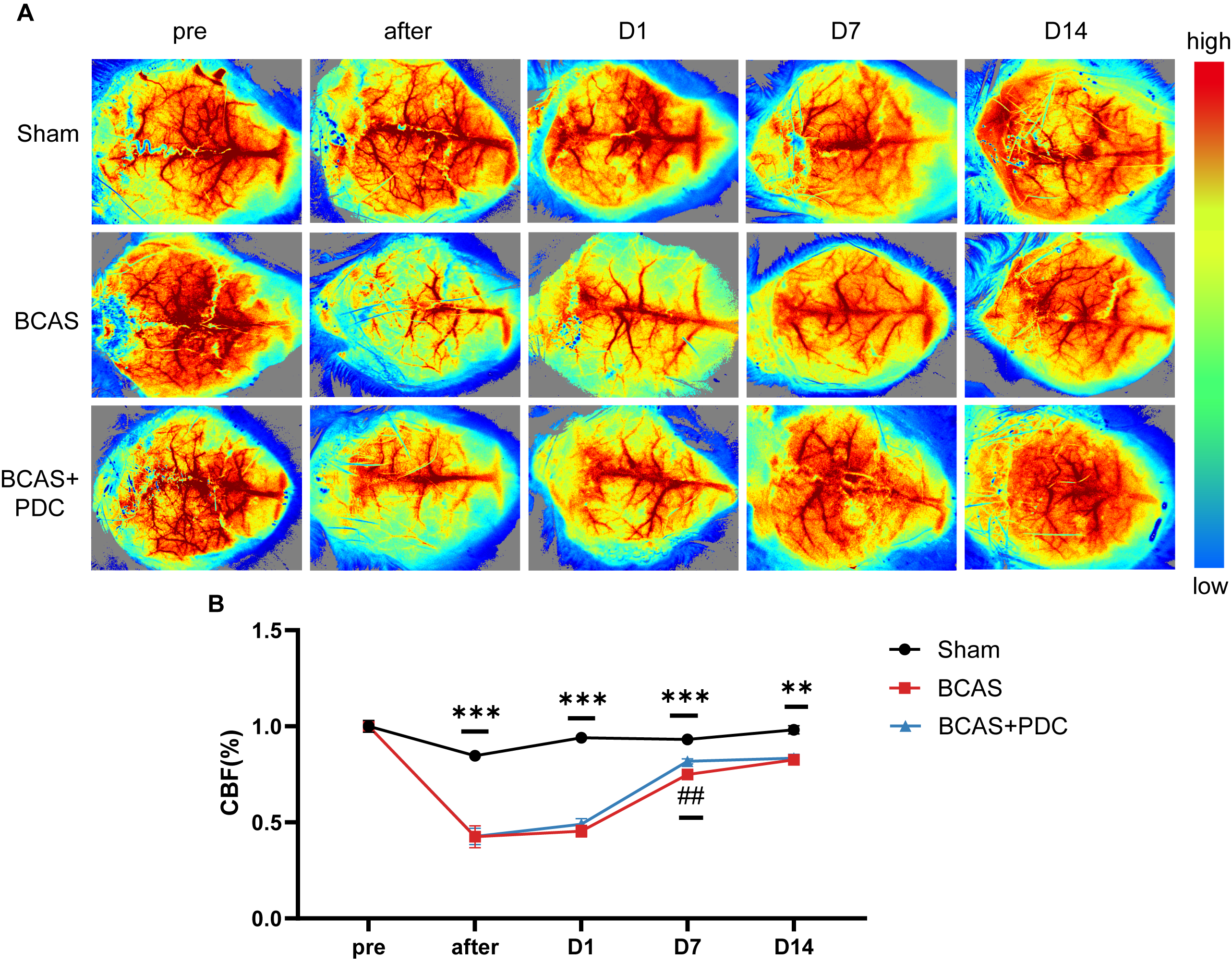

Supplement: Supplementary file 3 — Figure S3 [file CNS-30-e14487-s003.tif]
