# Supplementary material for: EAAT3 impedes oligodendrocyte remyelination in chronic cerebral hypoperfusion‐induced white matter injury
Source: CNS Neurosci Ther. 2023 Oct 6;30(1):e14487. doi: 10.1111/cns.14487 (PMC10805396; doi:10.1111/cns.14487)
Supplement: Supplementary file 4 — Figure S4 [file CNS-30-e14487-s002.pdf]

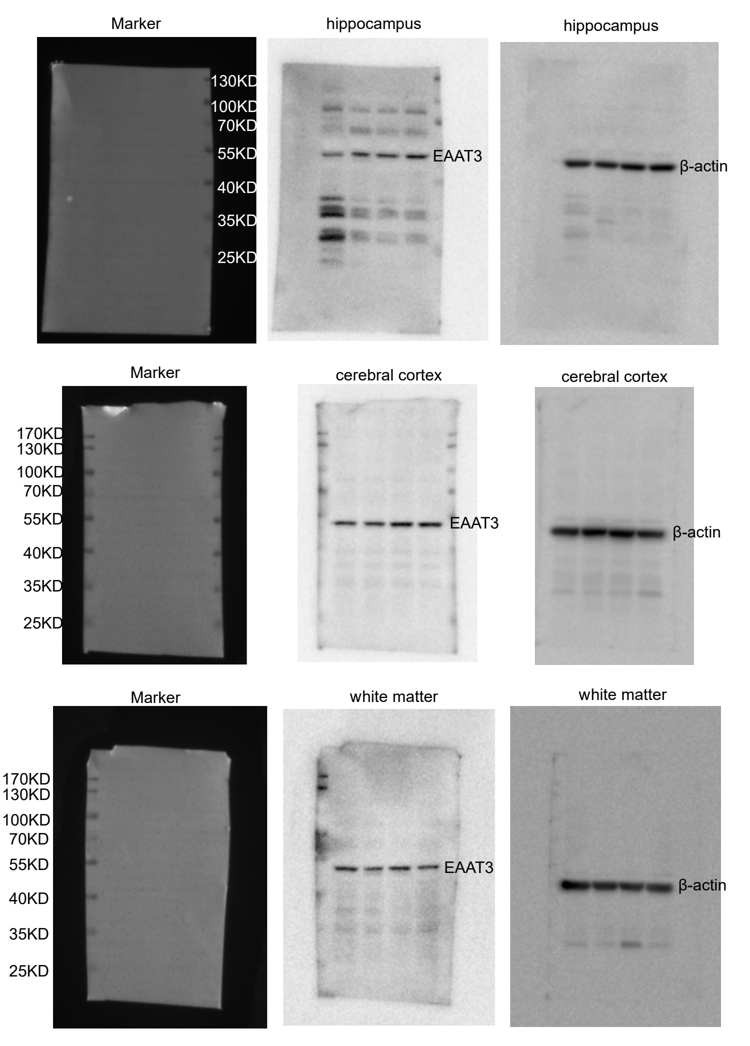

Full unedited gel/blot for Figure 1E in the manuscript

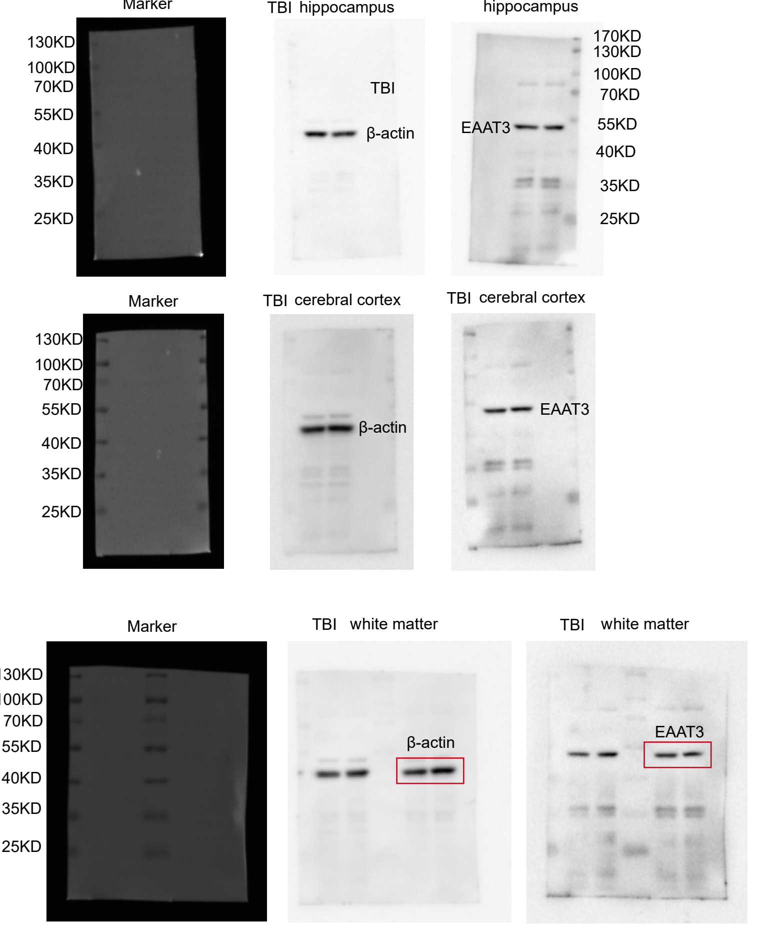

Full unedited gel/blot for Figure 1I in the manuscript

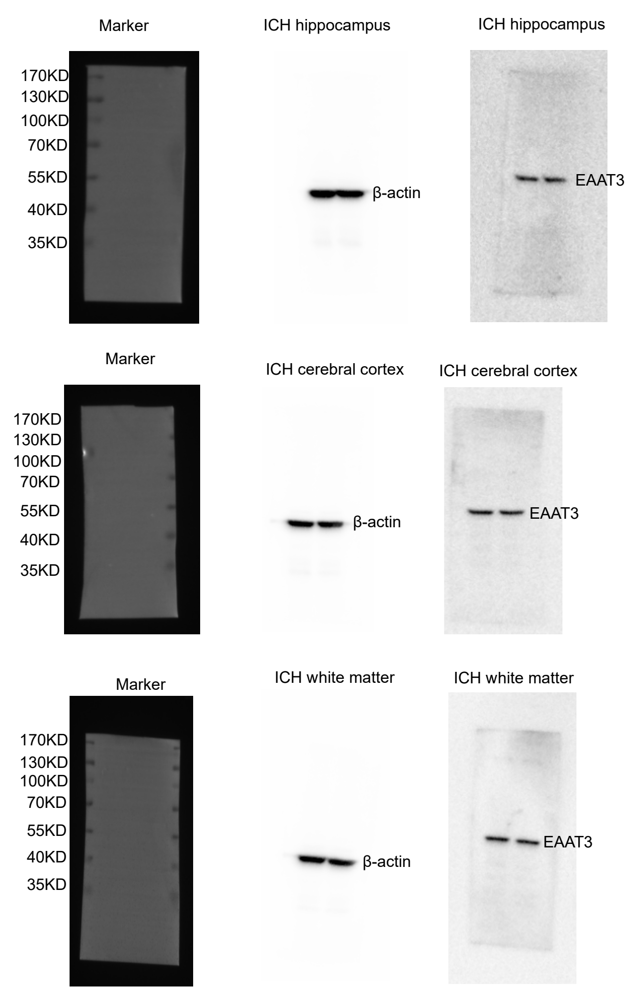

Full unedited gel/blot for Figure 1I in the manuscript

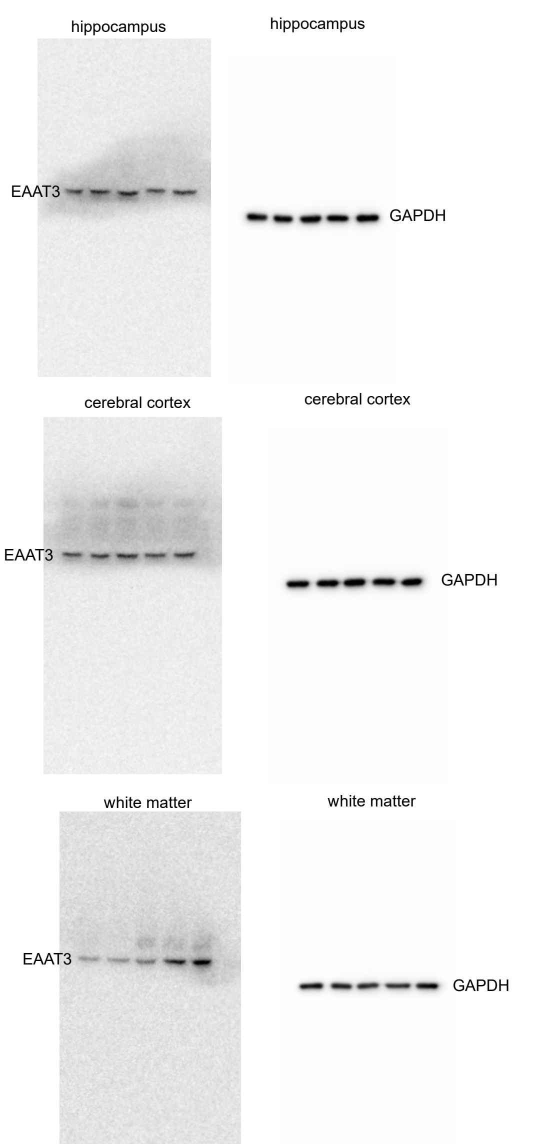

Full unedited gel/blot for Figure 1L in the manuscript

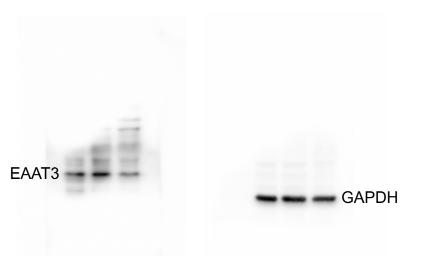

Full unedited gel/blot for Figure 2A in the manuscript

Full unedited gel/blot for Figure 2C in the manuscript

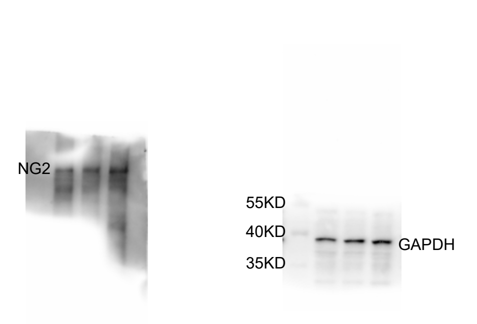

Full unedited gel/blot for Figure 3A in the manuscript

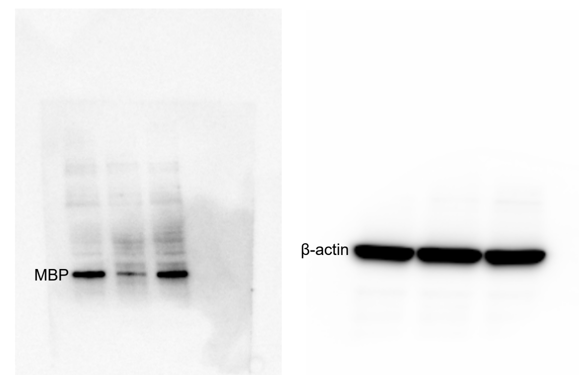

Full unedited gel/blot for Figure 4D in the manuscript

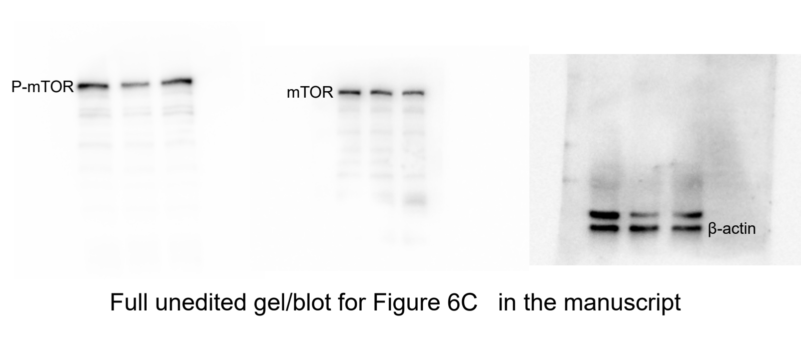

Full unedited gel/blot for Figure 6C in the manuscript

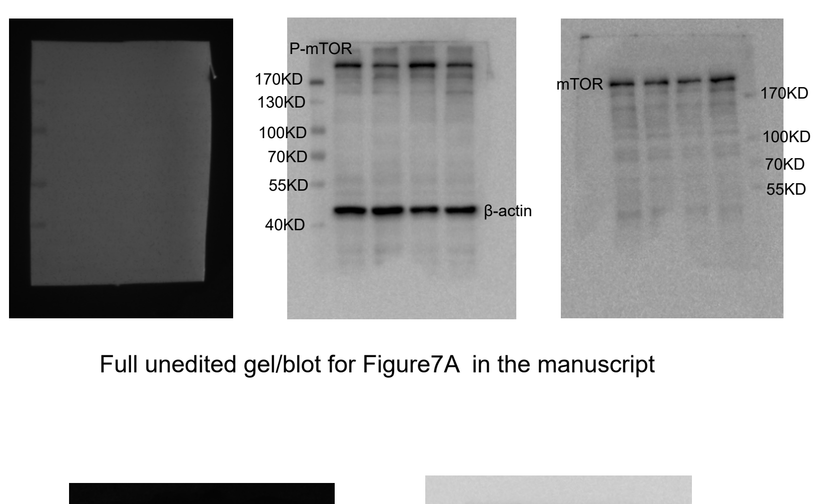

Full unedited gel/blot for Figure7A in the manuscript

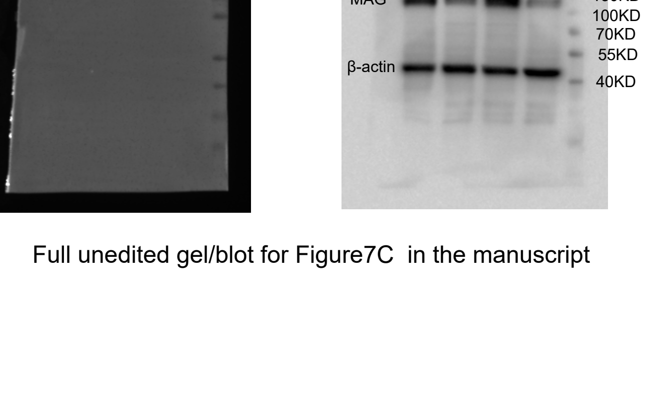

Full unedited gel/blot for Figure7C in the manuscript

Full unedited gel/blot for Supplementary Figure1B in the manuscript

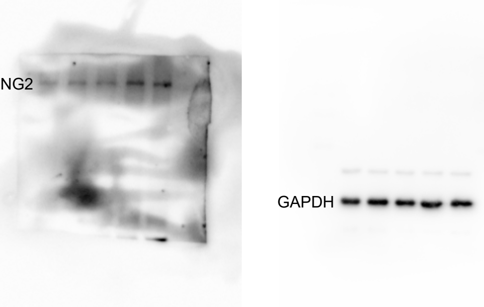

Full unedited gel/blot for Supplementary Figure2C in the manuscript
